# Functional and distinct roles of Piezo2-mediated mechanotransduction in dental primary afferent neurons

**DOI:** 10.1038/s41368-025-00374-8

**Published:** 2025-06-03

**Authors:** Pa Reum Lee, Kihwan Lee, Ji Min Park, Shinae Kim, Seog Bae Oh

**Affiliations:** 1 Department of Neurobiology and Physiology, School of Dentistry and Dental Research Institute, Seoul National University, Seoul, Republic of Korea; 2 Brain Science Institute, Korea Institute of Science and Technology, Seoul, Republic of Korea; 3 Tooth-Periodontium Complex Medical Research Center, Seoul National University, Seoul, Republic of Korea; 4 ADA Forsyth Institute, Cambridge, MA USA

**Keywords:** Ion channel signalling, Physiology

## Abstract

Piezo2, a mechanosensitive ion channel, serves as a crucial mechanotransducer in dental primary afferent (DPA) neurons and is potentially involved in hypersensitivity to mild mechanical irritations observed in dental patients. Given Piezo2’s widespread expression across diverse subpopulations of DPA neurons, this study aimed to characterize the mechanosensory properties of Piezo2-expressing DPA neurons with a focus on distinct features of voltage-gated sodium channels (VGSCs) and neuropeptide profiles. Using whole-cell patch-clamp recordings, we observed mechanically activated action potentials (APs) and classified AP waveforms based on the presence or absence of a hump during the repolarization phase. Single-cell reverse transcription polymerase chain reaction combined with patch-clamp recordings revealed specific associations between AP waveforms and molecular properties, including tetrodotoxin-resistant VGSCs (Na_V_1.8 and Na_V_1.9) and TRPV1 expression. Reanalysis of the transcriptomic dataset of DPA neurons identified correlations between neuropeptides—including two CGRP isoforms (α-CGRP and β-CGRP), Substance P, and Galanin—and the expression of Na_V_1.8 and Na_V_1.9, which were linked to defined AP subtypes. These molecular associations were further validated in Piezo2^+^ DPA neurons using fluorescence in situ hybridization. Together, these findings highlight the electrophysiological and neurochemical heterogeneity of Piezo2-expressing DPA neurons and their specialized roles in distinct mechanosensory signal transmission.

## Introduction

The high prevalence of pain in dental patients is often attributed to mild mechanical irritations, such as breathing, brushing, air puffs, and water spray directed at exposed dentin, rather than to noxious thermal stimuli like extreme heat or cold.^1–3^ These frequent clinical observations raise questions about the mechanisms underlying dentinal pain initiation, since weak mechanical stimuli like breathing or air puffs are typically insufficient to activate nociceptive afferents.^3^ Previous studies have demonstrated the intrinsic innervation of dental sensory nerves into the outermost pulp chamber, showing that most dentinal afferents are A-fibers with medium to large cell bodies and low-threshold mechanoreceptors (LTMRs).^3–5^ These specific types of tooth-innervating neurons, referred to as low-threshold algoneurons, are uniquely characterized by the co-expression of neurofilament 200 kDa (NF200), a marker of myelinated Aβ-fibers, and nociceptive neurotransmitters, including calcitonin gene-related peptide (CGRP), substance P, and neuropeptide Y.^3^ Algoneurons are suggested to intrinsically possess the properties of non-nociceptive A-fibers while also being capable of the synaptic transmission of pain sensations. Therefore, further study is needed to identify the specific mechanotransducers involved in this process in DPA neurons.

Piezo2, a major low-threshold mechanosensitive ion channel, plays a critical role in detecting gentle touch, stretch, vibration, and proprioception across various mammalian sensory organs.^6^ Piezo2 is abundant in sensory neurons, predominantly in large myelinated Aβ-fibers of the dorsal root ganglion (DRG), which are low-threshold, rapidly adapting (RA) fibers associated with light touch sensation.^7,8^ Several studies have reported that Piezo2 mediates mechanical allodynia (Aδ or Aβ pain) in nociceptive afferents;^9,10^ however, Piezo2’s role in mechanical pain remains controversial and appears to vary depending on in vivo models and pathological conditions.^9–11^ Unlike DRG neurons, Piezo2 activation in trigeminal ganglion (TG) neurons has been primarily studied in relation to pain responses induced by weak mechanical stimulation. In corneal afferent neurons, Piezo2 is functionally expressed in nociceptive populations and plays a role in discomfort and pain associated with dry eye and other ocular surface pathologies.^12^ Piezo2 is also broadly observed in DPA neurons, mostly in medium- to large-sized mechanosensitive neurons representing low-threshold algoneurons.^4,13,14^ Piezo2-expressing DPA neurons not only co-express NF200 and CGRP but also show high co-expression of the voltage-gated sodium channel (VGSC) Na_V_1.8,^13^ which contributes to the rising phase of action potentials (APs) and is predominantly found in nociceptive DRG neurons,^15^ or vesicular glutamate transporters (VGLUT1 and VGLUT2) involved in the glutamate signaling in primary afferents.^14^ In addition, DPA neurons display mechanosensitive RA currents in response to mechanical stimulation via Piezo2 activation.^13^ These findings indicate that DPA neurons include a specialized subpopulation involved in the nociceptive transmission of low-threshold mechanical stimuli, supporting Piezo2’s role as a potential transducer of mechanical stimuli in DPA neurons.^4,13,14^ Moreover, our recent study conducting single-cell RNA sequencing (scRNA-Seq) to classify DPA neuron subtypes has revealed widespread expression of *Piezo2* transcripts across multiple subtypes of DPA neurons.^16^ These findings raise further questions regarding whether the functional electrophysiological properties of Piezo2-expressing mechanosensitive DPA neurons are distinct.

In this study, we performed immunohistochemistry, whole-cell patch-clamp recordings, single-cell reverse transcription polymerase chain reaction (scRT-PCR), re-analysis of DPA neuron transcriptomic data,^16^ and fluorescence in situ hybridization (FISH) to uncover differential electrophysiological and neurochemical properties across DPA neurons, marked by Piezo2 expression. We focused on key features related to nerve signal transmission, specifically VGSCs and neuropeptide content. Together, our study provides a neurobiological understanding of how DPA neurons can differentially transmit mechanical stimulation via Piezo2 activation.

## Results

### Piezo2 is highly expressed in a subpopulation of DPA neurons

**Fig. 1:**
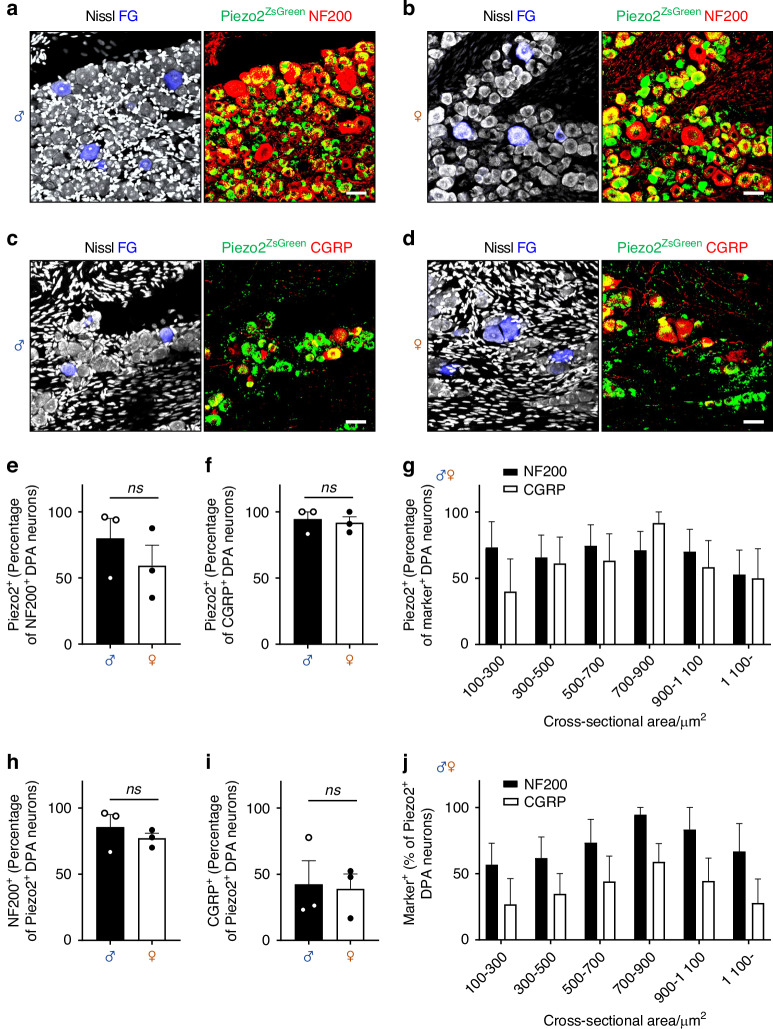
Piezo2 is highly expressed in a subset of DPA neurons that co-express NF200 and CGRP. **a**, **b** Representative images showing FG-labeled DPA neurons (blue) alongside Nissl-stained somata (white), with Piezo2 (green) and NF200 (red) in the maxillary region of the TG from male and female Piezo2-ZsGreen mice. *n* = 3 mice per gender. Scale bar: 40 μm. **c**, **d** Representative images depicting FG-labeled DPA neurons (blue) alongside Nissl-stained somata (white), with Piezo2 (green) and CGRP (red) in the maxillary region of the TG from male and female Piezo2-ZsGreen mice. Scale bar: 40 μm. **e**, **f** Proportion of Piezo2^+^ DPA neurons among NF200^+^ DPA neurons or among CGRP^+^ DPA neurons. **g** Cell body size distribution corresponding to panels (**e**, **f**). **h**, **i** Among Piezo2^+^ DPA neurons, proportion of NF200^+^ DPA neurons or CGRP^+^ DPA neurons. **j** Cell body size distribution corresponding to panels (**h**, **i**). The average of three sections per mouse is represented by the *n* number. Each NF200 and CGRP analysis used data from 3 mice per gender, while Piezo2 analysis used data from the same 6 mice per gender, simultaneously stained with NF200 or CGRP for panels (**g**, **j**). Data are shown as mean ± SEM

Previous studies have shown that Piezo2 is extensively expressed in the tooth-innervating afferents of rodent and human dental pulp.^13,14^ *Piezo2* mRNA transcripts in DPA neurons significantly overlap with markers for large myelinated sensory neurons, such as *Nefh* (NF200) and *S100b* (S100 Calcium Binding Protein B; S100B), as well as with a subpopulation that highly expresses *Calca* (CGRP).^4,13^ To specifically identify the subset of DPA neurons expressing Piezo2, we generated Piezo2-ZsGreen mice *(*Piezo2-Cre x Rosa26-ZsGreen). We investigated co-expression of Piezo2 with NF200 and CGRP in both male and female Piezo2-ZsGreen mice. Immunohistochemical analysis revealed Piezo2 expression in DPA neurons of various sizes marked with the retrograde neuronal tracer Fluoro-Gold (FG) (Fig. 1a–d). On average, Piezo2 was co-expressed in over 50% of NF200^+^ DPA neurons, and over 90% of CGRP^+^ neurons in both sexes (Fig. 1e, f). Conversely, among Piezo2^+^ DPA neurons, NF200 was expressed in over 70%, while CGRP expression was observed in less than 50% (Fig. 1h, i). No significant sex differences were observed for any marker (Fig. 1e, f, h, i). Further analysis of the size distribution of Piezo2-positive populations co-expressing NF200 and CGRP revealed the highest prevalence in medium- to large-sized DPA neurons (approximately 700 µm² to 900 µm², Fig. 1g, j), consistent with previous studies on the neurochemical characteristics of algoneuron-like DPA neurons expressing Piezo2.^13,16^

### Piezo2 activation elicits APs in DPA neurons

**Fig. 2:**
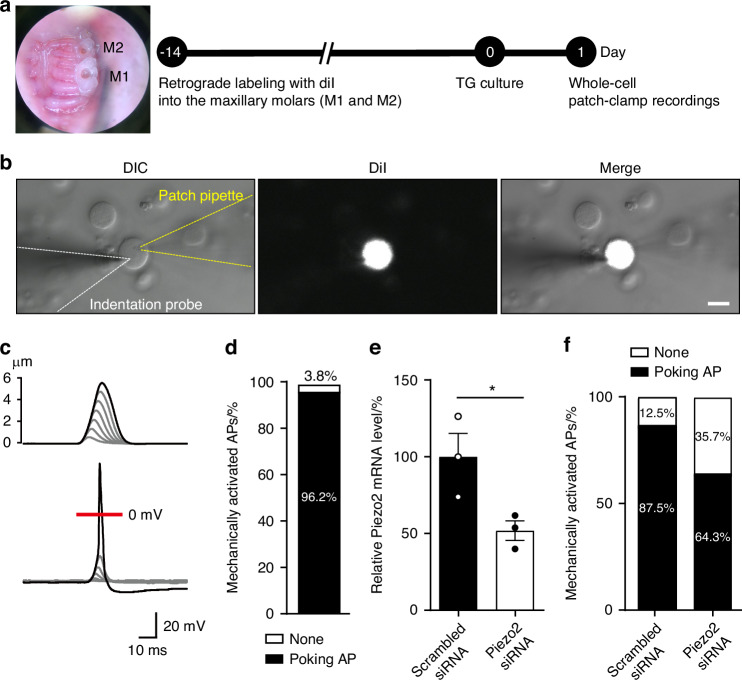
Poking stimulation induces APs via Piezo2 activation in DPA neurons. **a** Photograph of the maxillary first and second molars in wild-type mice undergoing labeling trials and schematic timeline for whole-cell patch-clamp recordings conducted 1 day after TG neuron culture. **b** Schematic of the poking stimulation setup (white dashed line: fire-polished glass pipette for poking stimulation; yellow dashed line: patch pipette for recordings). Images show DiI-labeled DPA neurons (white) as part of primary cultured TG neurons. Scale bar: 20 μm. **c** Representative current-clamp response to a series of poking stimuli incremented in 1 μm steps (upper traces) in DPA neurons. Sweeps applied every 20 s. The relaxation time constant of this current was 0.1 ms. The red line indicates the 0 mV level. **d** Ratio of mechanically activated APs in DPA neurons. Numbers in (or above) bars represent the percentage of neurons. *n* = 52 neurons from 10 mice. **e** Comparison of Piezo2 mRNA levels after 24 h of transfection with scrambled siRNA or Piezo2 siRNA in TG neurons. *n* = 3 mice for both groups. Unpaired Student’s *t* test: *P = 0.043. Data are shown as mean ± SEM. **f** The proportion of mechanically activated APs in DPA neurons transfected with scrambled siRNA or Piezo2 siRNA. Patch-clamp recordings were conducted with the experimenter blinded to the siRNA treatment. Numbers in bars represent the percentage of neurons. *n* = 6 mice for scrambled siRNA and *n* = 7 mice for Piezo2 siRNA

We labeled DPA neurons using a retrograde neuronal tracer DiI in both maxillary first and second molars of wild-type mice and then cultured TG neurons (Fig. 2a), including DPA neurons that distribute exclusively to the maxillary region of the TG (Fig. 1a–d). Activation of Piezo2 induces cation influx, leading to membrane depolarization and triggering AP generation via VGSCs.^17,18^ To examine whether DPA neurons follow this process, we conducted whole-cell patch-clamp recordings using poking stimulation in DiI-labeled DPA neurons to assess the generation of mechanically activated (MA) APs (Fig. 2b). We found that gradual increments in poking steps resulted in significant membrane depolarization, ultimately leading to the generation of an AP (Fig. 2c). Remarkably, 96.2% of DPA neurons exhibited MA APs (*n* = 50/52 neurons, Fig. 2d). To validate whether the APs were mediated by Piezo2, we applied knockdown to DPA neurons using scrambled siRNA or Piezo2 siRNA, as reported in a previous study.^13^ We confirmed that transfection with Piezo2 siRNA significantly reduced Piezo2 mRNA levels in TG neurons, compared to transfection of scrambled siRNA (Fig. 2e). Following siRNA transfection in DPA neurons, we observed that the firing ratio of APs was reduced in the Piezo2 siRNA-transfected DPA neurons (64.3%, *n* = 9/14 neurons) compared to scrambled siRNA-transfected DPA neurons (87.5%, *n* = 7/8 neurons, Fig. 2f), thus confirming the functional role of Piezo2 in generating MA APs in DPA neurons.

### Mechanosensitive Piezo2^+^ DPA neurons display two distinct AP waveforms

**Fig. 3:**
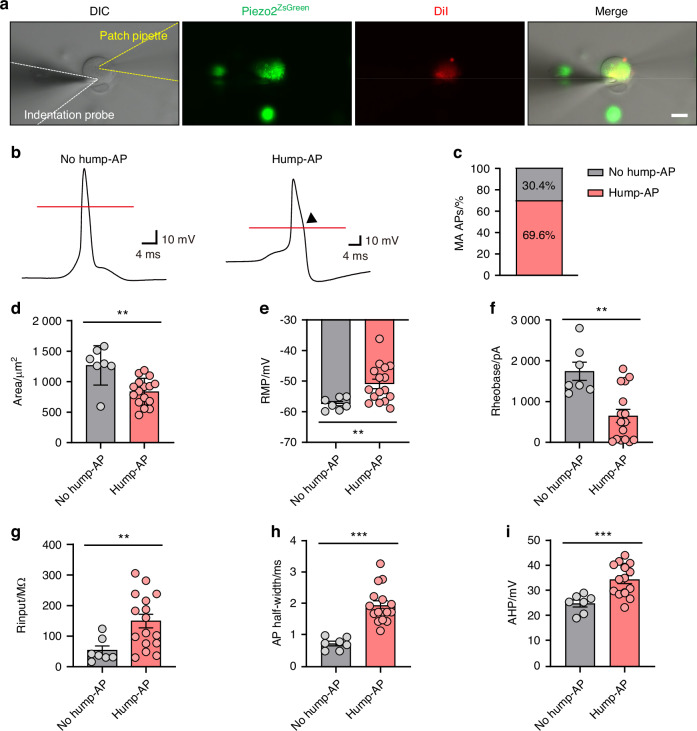
Electrophysiological properties of Piezo2-ZsGreen^+^ DPA neurons representing two distinct AP properties. **a** Schematic of the mechanical stimulation setup (white dashed line: fire-polished glass pipette for poking stimulation; yellow dashed line: patch pipette for recordings). Images show Piezo2-ZsGreen^+^ (green) and DiI-labeled (red) DPA neurons in primary cultured TG neurons from Piezo2-ZsGreen mice. Scale bar: 20 μm. **b** Representative traces of APs lacking a hump (no hump-AP) and with a hump (hump-AP) in current-clamp responses evoked by a series of poking stimuli in Piezo2-ZsGreen^+^ DPA neurons. The red lines indicate the 0 mV level. An arrowhead indicates the presence of a hump in the repolarization phase of the AP. **c** Proportion of mechanically activated (MA) APs in Piezo2-ZsGreen^+^ DPA neurons. The numbers in the bars represent the percentage of neurons. **d–i** Electrophysiological properties of Piezo2-ZsGreen^+^ DPA neurons displaying two distinct AP types. Bar graphs compare no hump-AP neurons and hump-AP neurons in terms of cell area (Mann–Whitney *U* test: **P = 0.003, **d**), resting membrane potentials (RMP, Welch’s *t* test: **P = 0.001, **e**), rheobase (Mann–Whitney *U* test: **P = 0.007, **f**), input resistance (Rinput, Unpaired Student’s *t* test: **P = 0.002, **g**), AP half-width (Unpaired Student’s *t* test: ***P < 0.001, **h**), afterhyperpolarization (AHP, Welch’s *t* test: ***P < 0.001, **i**). *n* = 7 neurons in the no hump-AP group and *n* = 16 neurons in the hump-AP group from 8 mice. Data are shown as mean ± SEM

AP shapes evoked by nerve stimulation have been proposed as sensory properties distinguishing nociceptive C-fibers from other neuronal types, including low-threshold mechanosensitive A-fibers.^19–21^ Previously, we identified two distinct AP shapes, in rat DPA neurons, designated type I and type II.^20^ Type I neurons exhibited broad APs with a hump in the repolarization phase, prolonged AP duration, responsiveness to capsaicin, which is an agonist of TRPV1 (transient receptor potential vanilloid 1), and smaller somata sizes. Conversely, type II neurons displayed APs lacking a hump, were insensitive to capsaicin, and had larger somata. Building on these findings, we investigated whether Piezo2^+^ DPA neurons exhibit different types of MA APs. Using Piezo2-ZsGreen mice, we cultured DPA neurons (Fig. 3a) and performed whole-cell patch-clamp recordings to examine AP shapes and electrophysiological intrinsic properties (Fig. 3b–i and Table 1). Piezo2-ZsGreen^+^ DPA neurons displayed two distinct AP shapes: APs lacking a hump (referred to as ‘no hump-AP’ neurons) and APs with a hump (referred to as ‘hump-AP’ neurons) (Fig. 3b). The proportion of hump-AP neurons (69.6%, *n* = 16/23 neurons) was higher than that of no hump-AP neurons (30.4%, *n* = 7/23 neurons, Fig. 3c). Furthermore, hump-AP neurons had smaller somata sizes compared to no hump-AP neurons (Fig. 3d), consistent with previous results.^19,20^ Interestingly, hump-AP neurons showed discrete electrophysiological characteristics compared to no hump-AP neurons (Fig. 3e–i). Specifically, hump-AP neurons showed more depolarized resting membrane potentials (RMP, Fig. 3e), reduced rheobase (the minimum current required to evoke an AP, Fig. 3f), increased input resistance (Fig. 3g), longer AP half-widths (Fig. 3h), and greater amplitudes of afterhyperpolarization (AHP, Fig. 3i), compared to no hump-AP neurons. However, no significant differences were observed between the two neuron types concerning maximum rise slope, depolarizing AP thresholds (the initial point on the AP’s rising phase), and AP amplitudes (Table 1). Our findings suggest that hump-AP neurons, characterized by alterations in RMP, rheobase, input resistance, and AP parameters, manifest heightened neuronal excitability compared to their no hump-AP counterparts.

**Table 1:** Physiological parameters of no hump-AP and hump-AP neurons

Physiological parameters	No hump-AP (*n* = 7)	Hump-AP (*n* = 16)	*P-*value	Analysis
Max dV/dT/(mV/ms)	344.2 ± 54.24	194.1 ± 21.23	0.639 7	Mann-Whitney *U*-test
Min dV/dT/(mV/ms)	-122.2 ± 10.43	-48.10 ± 2.612	0.000 3	Independent *t*-test
AP threshold/mV	-8.047 ± 5.813	-11.61 ± 3.272	0.385 1	Mann-Whitney *U*-test
AP amplitude/mV	69.73 ± 6.518	71.04 ± 3.055	0.836 8	Independent *t*-test

### Hump-AP neurons are associated with the expression of TTX-resistant VGSC isoforms

The observation of distinct MA AP types in Piezo2^+^ DPA neurons prompted further investigation into the expression of VGSC subtypes in these neurons. In mammalian adult sensory neurons, Na_V_1.1, Na_V_1.6, Na_V_1.7, Na_V_1.8, and Na_V_1.9 are well-established VGSC isoforms that can be classified based on their sensitivity to tetrodotoxin (TTX), which is a VGSC blocker, as either TTX-sensitive (TTX-S) or TTX-resistant (TTX-R) VGSCs.^15,22^ TTX-S VGSCs mainly include Na_V_1.1, Na_V_1.6, and Na_V_1.7, while TTX-R VGSCs, Na_V_1.8 and Na_V_1.9 are primarily expressed in sensory neurons typically associated with nociceptive functions.^22–24^ To investigate the neurochemical properties of distinct mechanosensitive DPA neurons, we performed scRT-PCR following whole-cell patch-clamp recordings in Piezo2-ZsGreen^+^ DPA neurons. The expression of VGSC isoforms, including *Scn1a* (Na_V_1.1), *Scn8a* (Na_V_1.6), *Scn9a* (Na_V_1.7), *Scn10a* (Na_V_1.8), and *Scn11a* (Na_V_1.9), was examined in recorded neurons (nested PCR primer information provided in Supplementary Table S1). Given that most *Scn1a*^+^ neurons co-express NF200,^15^ we hypothesized that *Scn1a* could serve as a marker gene for no hump-AP neurons. Moreover, since previous studies have reported a correlation between DPA neurons with hump-APs and TRPV1 expression,^20,21^ we also examined *Trpv1* expression in Piezo2^+^ DPA neurons. To further distinguish these neurons from C-LTMRs, we examined the expression of *Th* (Tyrosine hydroxylase), a representative marker of C-LTMRs.^25^

**Fig. 4:**
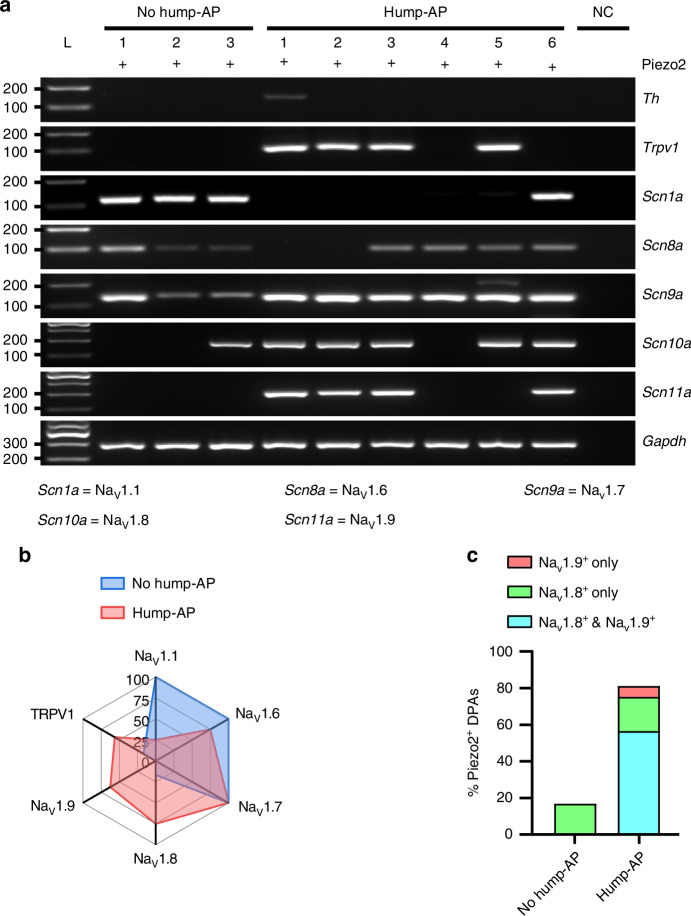
AP waveform-dependent molecular profiles in Piezo2-ZsGreen^+^ DPA neurons. **a** Representative gel images showing scRT-PCR products from no hump-AP and hump-AP in Piezo2-ZsGreen^+^ DPA neurons. Predicted sizes for selected markers: *Th* (Tyrosine hydroxylase): 152 bp, *Trpv1* (TRPV1): 115 bp, *Scn1a* (Na_V_1.6): 123 bp, *Scn8a* (Na_V_1.6): 97 bp, *Scn9a* (Na_V_1.7): 144 bp, *Scn10a* (Na_V_1.8): 190 bp, *Scn11a* (Na_V_1.9): 209 bp, and *Gapdh*: 282 bp. Negative control (NC) refers to a sample of bath solution. Gene names are indicated by their *gene symbols*. *n* = 6 neurons in the no hump-AP and *n* = 16 neurons in the hump-AP from 8 mice (scRT-PCR was performed on samples that underwent patch-clamp recordings in Fig. 3a–i). **b** Radar plots summarizing the proportion of each gene’s expression in no hump-AP and hump-AP Piezo2-ZsGreen^+^ DPA neurons, respectively. **c** Summary of scRT-PCR results of Na_V_1.8 and Na_V_1.9 in no hump-AP or hump-AP Piezo2-ZsGreen^+^ DPA neurons

Piezo2-ZsGreen^+^ DPA neurons rarely expressed *Th* mRNA (no hump-AP: 0%, *n* = 0/6 neurons; hump-AP: 12.5%, *n* = 2/16 neurons, Fig. 4a), suggesting that most of them correspond to A-LTMRs.^3,4,13^ *Trpv1* was distinctly expressed in hump-AP neurons (56.3%, *n* = 9/16 neurons) compared to no hump-AP neurons (14.3%, *n* = 1/6 neurons, Fig. 4a, b). Interestingly, *Scn1a* was predominant in no hump-AP neurons (no hump-AP: 100%, *n* = 6/6 neurons; hump-AP: 25%, *n* = 4/16 neurons), while Na_V_1.6 and Na_V_1.7 were highly expressed in both no hump-AP neurons (Na_V_1.6: 100%, *n* = 6/6 neurons; Na_V_1.7: 100%, *n* = 6/6 neurons) and hump-AP neurons (Na_V_1.6: 75%, *n* = 12/16 neurons; Na_V_1.7: 100%, *n* = 16/16 neurons, Fig. 4a, b). However, Na_V_1.8 and Na_V_1.9 exhibited significantly higher expression levels in hump-AP neurons (Na_V_1.8: 75%, *n* = 12/16 neurons; Na_V_1.9: 62.5%, *n* = 10/16 neurons) compared to no hump-AP neurons (Na_V_1.8: 16.7%, *n* = 1/6 neurons, Na_V_1.9: 0%, *n* = 0/6 neurons, Fig. 4a, b). As Na_V_1.9 was exclusively expressed in hump-AP neurons, all neurons co-expressing Na_V_1.8 with Na_V_1.9 were found in hump-AP neurons (56.3%, *n* = 9/16 neurons, Fig. 4c). Additionally, the single expression of Na_V_1.8 was comparable between no hump-AP neurons (16.7%, *n* = 1/6 neurons) and hump-AP neurons (18.8%, *n* = 3/16 neurons, Fig. 4c). Collectively, our scRT-PCR analysis revealed that the co-localization of Na_V_1.8 and Na_V_1.9 was more strongly associated with this neuronal subtype, although TRPV1 exhibited moderate co-expression with hump-AP Piezo2^+^ DPA neurons.

**Fig. 5:**
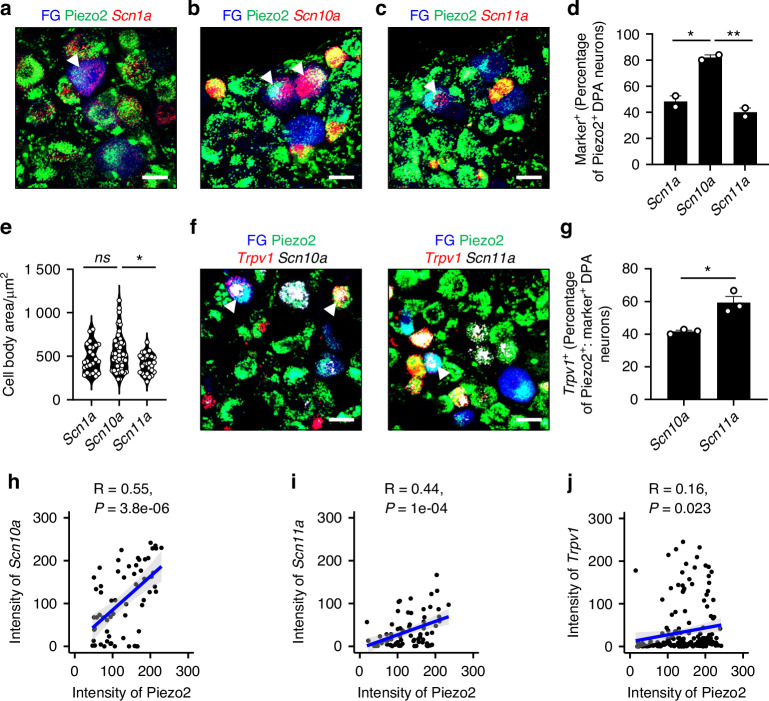
Co-expression and correlation patterns of VGSC isoforms and TRPV1 in Piezo2-ZsGreen^+^ DPA neurons. **a–c** Representative z-stacked confocal images showing *Scn1a*, *Scn10a*, and *Scn11a* mRNAs (red) in FG-labeled DPA neurons (blue) expressing Piezo2 (green). White arrowheads mark double-positive DPA neurons. Scale bars: 20 μm. **d** Proportion of Piezo2^+^ DPA neurons that express each marker gene. *n* = 2 mice for all groups. One-way ANOVA followed by Bonferroni’s multiple comparisons test: *P = 0.016 4, **P = 0.008 7. **e** Violin plots representing the cell body size distribution (µm^2^) of marker^+^ neurons (*Scn1a*: *n* = 25 neurons; *Scn10a*: *n* = 50 neurons; *Scn11a*: *n* = 27 neurons). Kruskal-Wallis followed by Dunn’s multiple comparisons test, *P = 0.029 3. **f** Representative z-stacked confocal images showing *Trpv1* mRNAs (red) in *Scn10*^+^ or *Scn11a*^+^ (white) FG-labeled DPA neurons (blue) expressing Piezo2 (green). White arrowheads mark triple-positive DPA neurons. Scale bars: 20 μm. **g** Comparative analysis of the proportion of *Trpv1*^+^ DPA neurons among Piezo2^+^ DPA neurons and each marker^+^ DPA neuron group. *n* = 3 mice for both groups. Unpaired Student’s *t* test: *P = 0.010 2. Data are shown as mean ± SEM. **h–j** Scatter plots displaying individual data points (black) from FG-labeled DPA neurons, with Pearson correlation coefficients (*R*), *P*-values, and fitted linear regression lines (blue) indicating the correlation trends between Piezo2 and *Scn10a*/*Scn11a*/*Trpv1*

To further investigate the molecularly distinct subpopulations of Piezo2^+^ DPA neurons, we examined the proportion of *Scn1a*, *Scn10a*, and *Scn11a* expression using FISH (Fig. 5a–e). On average*, Scn10a* was the most frequently co-expressed gene in Piezo2^+^ DPA neurons, showing a significantly higher proportion than *Scn1a* and *Scn11a* (Fig. 5d). Furthermore, an analysis of cell body size in gene-positive neurons (Fig. 5d) revealed that *Scn11a*^+^ Piezo2^+^ DPA neurons were smaller than other subpopulations (Fig. 5e). Additionally, *Trpv1* was more highly co-expressed with *Scn11a* than with *Scn10a* in Piezo2^+^ DPA neurons (Fig. 5f, g), suggesting that Na_V_1.9 may contribute to signal transduction in heat- and mechanically-sensitive DPA neurons. To assess correlations between gene expression levels, we quantified the mean fluorescence intensity values of ion channels expressed in FG-labeled DPA neurons from FISH images (Fig. 5b, c, f). Pearson correlation analysis revealed a moderate-to-strong positive correlation between Piezo2 and *Scn10a* expression levels (R = 0.55, *P* = 3.8e-06, Fig. 5h) and a moderate correlation between Piezo2 and *Scn11a* (R = 0.44, *P* = 1e-04, Fig. 5i), whereas *Trpv1* showed only a weak correlation with Piezo2 (R = 0.16, *P* = 0.023, Fig. 5j). These results suggest that Piezo2 expression levels in most DPA neurons are more closely associated with *Scn10a* and *Scn11a* than with *Trpv1*. However, correlation analysis alone does not establish a direct functional interaction, so further investigation is required to determine whether their spatial associations contribute to Piezo2-mediated mechanosensory signaling.

**Fig. 6:**
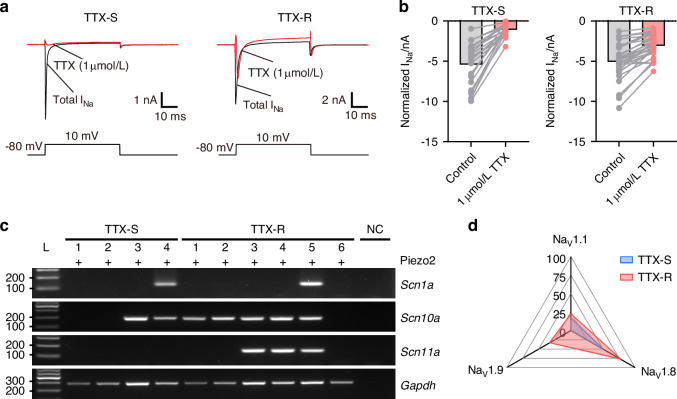
Distinct subtypes identified based on hump-AP and TTX-R VGSCs in Piezo2-ZsGreen^+^ DPA neurons. **a** Representative Na^+^ currents (*I*_Na_) traces of TTX-S (left) and TTX-R (right) Piezo2^+^ DPA neurons. Black lines indicate total current traces, and red lines indicate remaining current traces following the application of 1 μmol/L TTX. All test pulses were applied for 50 ms at +10 mV, with a holding potential of −80 mV. **b** Bar graphs showing significantly different sodium current inhibition in TTX-S neurons (*n* = 21), compared to TTX-R neurons (*n* = 31) in Piezo2-ZsGreen^+^ DPA neurons. *n* = 9 mice. **c** Representative gel images showing scRT-PCR products from TTX-S and TTX-R Piezo2-ZsGreen^+^ DPA neurons. Predicted sizes for selected markers: *Scn1a* (Na_V_1.6): 123 bp, *Scn10a* (Na_V_1.8): 190 bp, *Scn11a* (Na_V_1.9): 209 bp, and *Gapdh*: 282 bp. Negative control (NC) refers to a sample of bath solution. *n* = 6 neurons in TTX-S and *n* = 9 neurons in TTX-R from 6 mice. **d** Radar plots summarizing the proportion of each gene expressed in TTX-S and TTX-R Piezo2-ZsGreen^+^ DPA neurons, respectively

We then investigated the functional classification of Piezo2^+^ DPA neurons by measuring *I*_*Na*_ through VGSCs in Piezo2-ZsGreen^+^ DPA neurons. We identified two distinct types present in Piezo2-ZsGreen^+^ DPA neurons: TTX-S neurons, which were completely inhibited by 1 μmol/L TTX, and TTX-R neurons, which retained *I*_*Na*_ even in the presence of 1 μmol/L TTX (Fig. 6a, b). To further validate the relationship between *Scn1a* (Na_V_1.1)*, Scn10a* (Na_V_1.8), and *Scn11a* (Na_V_1.9) expression and *I*_*Na*_ properties, scRT-PCR was performed following whole-cell patch-clamp recordings (Fig. 6c, d). Na_V_1.1 expression was similarly low in both TTX-S and TTX-R neurons (TTX-S: 16.7%, *n* = 1/6 neurons; TTX-R: 22.2%, *n* = 2/9 neurons, Fig. 6d). In contrast, Na_V_1.8 expression was higher in TTX-R neurons than in TTX-S neurons (TTX-S: 50%, *n* = 3/6 neurons; TTX-R: 77.8%, *n* = 7/9 neurons, Fig. 6d). Interestingly, although Na_V_1.9 expression was not highly prevalent, it was exclusively detected in TTX-R neurons under our experimental conditions (TTX-S: 0%, *n* = 0/6 neurons; TTX-R: 33.3%, *n* = 3/9 neurons, Fig. 6d). Taken together, the co-expression of Na_V_1.8 and Na_V_1.9 in Piezo2^+^ DPA neurons functionally classifies them as TTX-R neurons, which correlate with hump-AP neurons due to the high expression of Na_V_1.8 and Na_V_1.9. These findings suggest that hump-AP neurons exhibit functional properties consistent with the transmission of mechanical nociceptive signals.

### Specific isoforms of VGSCs in Piezo2^+^ DPA neurons exhibit distinct neuropeptide expression patterns

The generation of APs in sensory neurons activates voltage-gated calcium channels (VGCCs), which in turn leads to the synaptic release of various signaling neuropeptides from sensory neuron terminals to the central nervous system.^26^ Having identified *Scn10a* and *Scn11a* as key marker genes for hump-AP and TTX-R neurons (Figs. 4–6), we next aimed to explore the neuropeptide profiles associated with their expression. To achieve this, we used a previously published scRNA-Seq dataset of DPA neurons.^16^ Neurons with Piezo2 expression (Piezo2 read counts > 0, approximately 88% of the total DPA neurons, *n* = 73/83 neurons) were selected and categorized into two groups based on *Scn10a* and *Scn11a* transcript levels: the negative group (*Scn10a* or *Scn11a* read counts = 0, 15.1% of Piezo2^+^ DPA neurons, *n* = 11/73 neurons) and the positive group (*Scn10a* and *Scn11a* read counts > 0, 84.9% of Piezo2^+^ DPA neurons, *n* = 62/73 neurons).

**Fig. 7:**
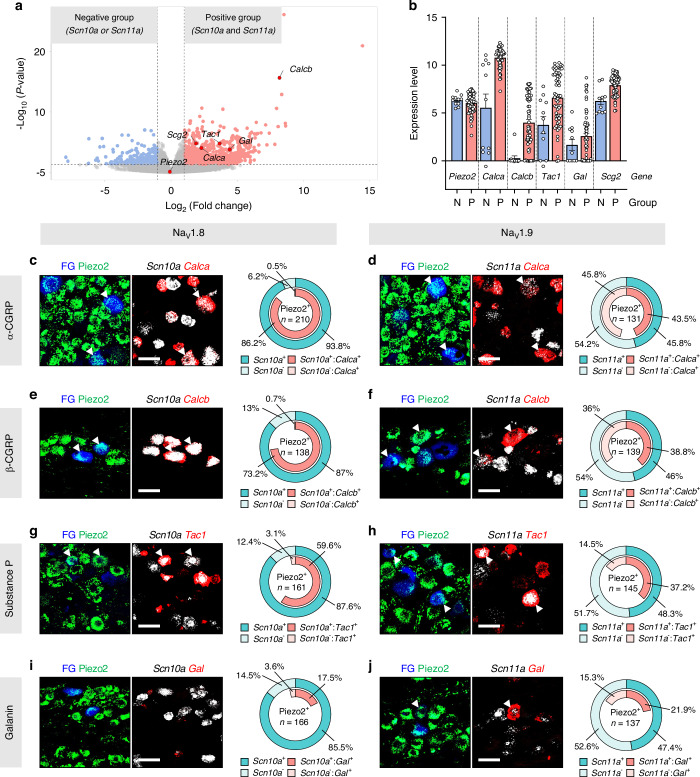
Neuropeptide expression in relation to Na_V_1.8 and Na_V_1.9 in Piezo2-ZsGreen^+^ DPA neurons. **a** Volcano plot depicting DE genes with a log_2_ fold change greater than 1.0 and an adjusted *P*-value less than 0.05 (indicated by dotted lines), enriched in either *Scn10a*^-^ or *Scn11a*^-^ neurons within the Piezo2^+^ DPA population (left side, designated as the negative group) or in *Scn10a*^+^ and *Scn11a*^+^ neurons within the Piezo2^+^ DPA population (right side, designated as the positive group). Genes of interest are labeled in the plot. Raw data are presented in Supplementary Tables S2 and S3. **b** Expression levels (log-normalized read counts) of neuropeptide-related genes meeting the above criteria in the DPA neuron transcriptome, including Piezo2, *Calca* (α-CGRP), *Calcb* (β-CGRP), *Tac1* (Substance P), *Gal* (Galanin), *and Scg2* (Secretogranin II). **c–j** Representative z-stacked confocal images showing *Calca*, *Calcb*, *Tac1*, and *Gal* mRNAs (red) in Piezo2^+^ FG-labeled DPA neurons (green and blue) co-expressing *Scn10a* or *Scn11a* mRNAs (white). White arrowheads mark triple-positive DPA neurons. Scale bars: 20 μm. The outer pie charts represent the proportion of Piezo2^+^ DPA neurons that express *Scn10a* or *Scn11a* and lack both makers. The inner pie charts illustrate the proportion of neurons expressing specific neuropeptide genes among DPA neurons that express *Scn10a* or *Scn11a* and lack both markers. *n* = 3 mice for all groups

As expected, differential expression (DE) analysis using DESeq2 revealed significantly higher expression of *Scn9a, Scn10a*, *Scn11a*, as well as *Trpv1*, in the positive group compared to the negative group (Supplementary Fig. S1a, b and Supplementary Table S3). Piezo2 expression was comparable between the two groups (Fig. 7a, b). When investigating neuropeptide-related genes, we identified *Calca* (α-CGRP), *Calcb* (β-CGRP), *Tac1* (Substance P), *Gal* (Galanin), and *Scg2* (Secretogranin II), which are known to be released from dental primary afferents and are implicated in inflammation and pain.^2,27–30^ These genes were significantly upregulated in the positive group compared to the negative group (Fig. 7a, b, and Supplementary Table S3). Notably, no genes associated with VGSCs or neuropeptides were upregulated in the negative group relative to the positive group (Fig. 7a and Supplementary Table S2). These results suggest that, in addition to CGRP, previously identified as a major molecule in nociceptive signaling,^3,13^ other potential neuropeptides may also participate in nociceptive signaling functions.

To validate our DE analysis, we employed FISH and scRT-PCR to examine the expression of *Calca*, *Calcb*, *Tac1*, and *Gal* in Piezo2-ZsGreen^+^ DPA neurons labeled with FG. Our results demonstrated significant upregulation of these neuropeptide genes in Piezo2^+^ DPA neurons co-expressing *Scn10a* and *Scn11a* (Fig. 7c–j). Strikingly, this upregulation was particularly pronounced for *Calca*, *Calcb*, and *Tac1* in correlation with *Scn10a* (Fig. 7c, e, g) compared to *Scn11a* (Fig. 7d, f, h), suggesting that these neuropeptides are predominantly released from Na_V_1.8-expressing nociceptive neurons. Furthermore, scRT-PCR revealed significantly elevated expression of *Calca*, *Calcb*, *Tac1*, and *Gal* in hump-AP neurons (*Calca*: 93.8%, *n* = 15/16 neurons; *Calcb*: 75%, *n* = 12/16 neurons; *Tac1*: 75%, *n* = 12/16 neurons; Gal: 50%, *n* = 8/16 neurons; Supplementary Fig. S2a, b) compared to no hump-AP neurons (*Calca*: 50%, *n* = 3/6 neurons; *Calcb*: 0%, *n* = 0/6 neurons; *Tac1*: 33.3%, *n* = 2/6 neurons; *Gal*: 16.7%, *n* = 1/6 neurons; Supplementary Fig. S2a, b), consistent with our FISH results. Therefore, our findings suggest that β-CGRP, alongside α-CGRP and substance P, may serve as highly specific neuropeptide markers for nociceptor-like Piezo2^+^ DPA neurons.

## Discussion

**Fig. 8:**
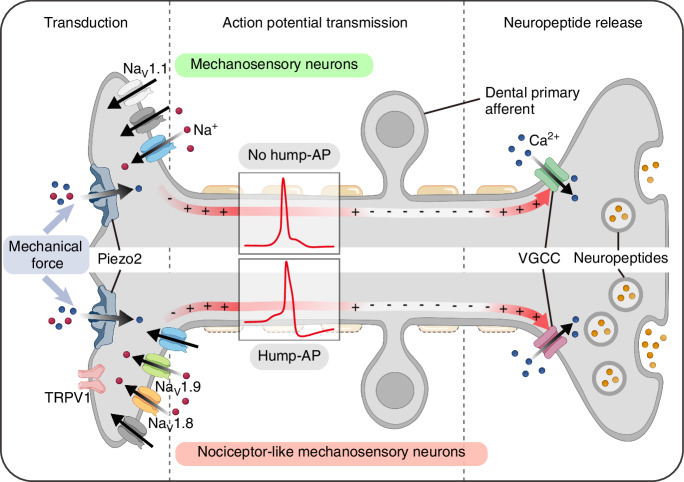
Schematic representation of the proposed model of Piezo2-mediated mechanotransduction in two distinct types of DPA neurons. Illustration was created with BioRender.com. Na_V_ channels voltage-gated sodium channels, TRPV1 transient receptor potential vanilloid 1, AP action potential, VGCC voltage-gated calcium channel

This study provides significant insights into Piezo2-mediated mechanosensory signaling in DPA neurons, revealing distinct functional and neurochemical subtypes. These subtypes were characterized by differences in Piezo2-mediated AP waveforms, TTX sensitivity, and the expression of key molecular markers, including TRPV1, VGSC isoforms, and neuropeptides. Based on these characterizations, we propose that Piezo2-mediated mechanotransduction activates distinct mechanosensory signaling pathways in two neuronal subtypes of DPA neurons: TTX-S mechanosensory neurons and TTX-R nociceptor-like mechanosensory neurons (summarized in Fig. 8). These pathways may involve subtype-specific modulation by nociceptive neuropeptides, including CGRP isoforms (α-CGRP and β-CGRP), Substance P, and Galanin, potentially driving distinct central signaling outcomes.

Our first findings demonstrated that Piezo2 is highly co-expressed in NF200^+^ and CGRP^+^ subpopulations of DPA neurons, which are likely to represent low-threshold algoneurons (Fig. 1a–j), consistent with previous studies.^3,13^ The majority of DPA neurons expressing Piezo2 generated APs in response to mechanical poking stimulation (Fig. 2a–d) and exhibited distinct AP properties related to electrogenesis and excitability, indicative of discrete functional subtypes within Piezo2^+^ DPA neurons (Fig. 3a–i). Wide APs with a hump during the repolarization phase were associated with the expression of TTX-R VGSCs, specifically Na_V_1.8 and Na_V_1.9, which are predominantly linked to nociceptive neurons.^15,19,24^ Na_V_1.8 facilitates overshooting APs at more depolarized steady-state potentials, and TTX-R sodium currents enhance calcium entry, thereby influencing calcium-activated potassium currents and shaping the humps in APs.^24,31^ Na_V_1.9 contributes to subthreshold depolarizations critical for AP electrogenesis and lowers the threshold for repetitive firing.^32,33^ Therefore, Piezo2 activation may transmit different signals through diverse AP properties in specific subpopulations, suggesting functionally distinct roles in mechanosensory signal transmission.

Given the critical roles of Na_V_1.8 and Na_V_1.9 in nociceptive sensory signaling, we examined whether the subpopulation of Piezo2^+^ DPA neurons displaying hump-AP waveforms shares characteristics with conventional nociceptors. By combining scRT-PCR with electrophysiological recordings, we uncovered compelling evidence linking distinct AP waveforms to the differential expression patterns of VGSC isoforms. Specifically, Na_V_1.1 was predominantly expressed in no hump-AP neurons, which likely represent non-nociceptive mechanosensory subtypes (Fig. 4a, b and Fig. 5a–e). In contrast, Na_V_1.8 and Na_V_1.9 were selectively expressed in hump-AP neurons, suggesting their potential role in mechanically evoked nociception (Fig. 4a–c and Fig. 5a–e). Notably, we found that TRPV1, previously proposed as a marker for hump-AP DPA neurons,^20^ exhibited a stronger association with Na_V_1.9 than with Na_V_1.8 (Fig. 5f, g). This observation suggests that polymodal DPA neurons, capable of responding to both heat and mechanical stimulation, may preferentially transmit mechanical signals through Na_V_1.9.^34^ Interestingly, our Pearson correlation analysis revealed that Piezo2 expression levels correlated more strongly with Na_V_1.8 and Na_V_1.9 than with TRPV1 (Fig. 5h–j). Given that Piezo2 activation induces membrane depolarization through cation influx,^17,18^ and that Na_V_1.8 and Na_V_1.9 facilitate AP generation and sustained excitability in nociceptors,^15^ the co-expression of these channels may synergistically enhance mechano-nociceptive signal transmission. We further confirmed that Piezo2^+^ DPA neurons with relatively high levels of Na_V_1.8 and Na_V_1.9 expression shares TTX-R properties (Fig. 6a–d), further supporting their classification as nociceptive neurons. Based on these findings, we concluded that Na_V_1.8 and Na_V_1.9 serve as reliable markers for nociceptor-like Piezo2^+^ DPA neurons characterized by hump-APs and TTX-R properties.

To further strengthen our findings, we investigated differences in neuropeptides involved in pain transmission using the DPA neuron transcriptome dataset.^16^ This analysis revealed a significant association between signaling neuropeptides—such as two CGRP isoforms (α-CGRP and β-CGRP), Substance P, and Galanin—and Piezo2^+^ DPA neurons that express Na_V_1.8 and Na_V_1.9 (Fig. 7a–b). Notably, both α-CGRP and β-CGRP were highly associated with nociceptor-like Piezo2^+^ DPA neurons, as confirmed by FISH and scRT-PCR results (Fig. 7c–j and Supplementary Fig. S2a, b). These findings align well with a previous report demonstrating colocalization of β-CGRP and substance P with α-CGRP in small to medium-sized DRG neurons, but not in large α-CGRP^+^ DRG neurons in rats.^35^ However, unlike α-CGRP, β-CGRP has been primarily associated with the enteric nervous system rather than the somatosensory system,^36,37^ indicating that more detailed studies are required to investigate its specific functional role in DPA neurons. Meanwhile, substance P is a well-established neuropeptide involved in neuroinflammation and inflammatory dental pain conditions, such as pulpitis.^2,38,39^ It is released from sensitized nociceptors and shares overlapping functional roles with CGRP.^40^ Galanin has also been reported to be upregulated in the TG following tooth movement,^28^ although its expression was very limited in DPA neurons under our experimental conditions (Fig. 7i, j). Similarly, secretogranin II, a gene activated by FOS that encodes the neuropeptide secretoneurin, has been identified in the sensory innervation of the human dental pulp, suggesting a role in neurogenic inflammation.^30^ However, since our gene search was based on gene ontology lists of previously reported neuropeptides, this study was limited in identifying novel or uncharacterized genes. Additionally, we found that among voltage-gated calcium channels (VGCCs), only *Cacna1e* (Ca_V_2.3) and *Cacna1h* (Ca_V_3.2) were significantly upregulated in nociceptor-like Piezo2^+^ DPA neurons (Supplementary Fig. S1a, c and Supplementary Table S3) and hump-AP neurons (Supplementary Fig. S1d, e). Both Ca_V_2.3 and Ca_V_3.2 channels are known to play critical roles in signal conductance along nociceptive neurons and have been identified as potential drug targets for analgesics.^41–43^ These findings further support the conclusion that Piezo2^+^ DPA neurons expressing Na_V_1.8 and Na_V_1.9 function as nociceptors.

Piezo channels are present in the outer layer of both human and mouse dental pulp, as well as in periodontal ligaments.^14,44^ This suggests that Piezo channels are expressed not only in pulpal afferents but also in various non-neuronal cells, such as dental pulp stem cells/pre-odontoblasts, odontoblasts, and periodontal ligament fibroblasts,^44^ where they play a crucial role in the detection of mechanical forces, an essential process for cellular functions including development, differentiation, proliferation, migration, and mechanosensation in diverse biological contexts.^44^ In various cell types within the pulp chamber, Piezo channels can be activated by internal mechanical forces, such as orthodontic tooth movement, cyclic tensile stress in periodontal ligaments, or changes in intrapulpal pressure.^14,44–46^ They can also respond to direct external mechanical stimuli, including brushing, air puffs, or dentinal fluid movement at exposed dentin.^13,14^

While Piezo channels in non-neuronal cells contribute to cellular mechanosensation and tissue homeostasis, Piezo2 in somatosensory neurons specifically functions as the primary mechanotransducer by converting mechanical stimuli into electrical signals for sensory transmission.^47,48^ Over the past decade, considerable progress has been made in understanding Piezo2-mediated mechanosensory signaling mechanisms in these neurons. Upon mechanical stimulation, Piezo2 channels facilitate cation influx, leading to membrane depolarization and subsequent activation of VGSCs.^47,48^ This local depolarization, known as the receptor potential, can evoke APs that propagate mechanical signals within neural circuits. Once the AP reaches the synaptic terminal, VGCCs are activated, leading to sufficient calcium influx to drive the release of neurotransmitters and neuropeptides, thereby facilitating mechanosensory transmission to higher-order processing centers. Beyond its role in AP generation, calcium entry through Piezo2 channels can also initiate intracellular signaling cascades involved in gene transcription, cytoskeletal remodeling, and bone homeostasis.^48–50^ Additionally, Piezo2 activity is modulated by lipid-based or cyclic nucleotide signaling molecules in a calcium-dependent manner.^51,52^ However, the precise mechanisms by which nociceptive mediators and intracellular signaling pathways regulate Piezo2-mediated mechanotransduction under pain conditions remain unclear, highlighting the need for further investigation by future studies.^48^

Our findings suggest that nociceptor-like Piezo2^+^ DPA neurons co-expressing moderate-to-high levels of TRPV1, Na_V_1.8, and Na_V_1.9 may serve as key molecular targets for modulating pain-related mechanotransduction (Figs. 4–7). Accordingly, pharmacological inhibition of Piezo2 activity in these neurons could be achieved using Piezo2-specific blockers such as GsMTx4^53^ or selective inhibitors targeting Na_V_1.8 and Na_V_1.9. Additionally, analgesic drugs such as QX-314, a lidocaine derivative that enters nociceptive neurons via activated TRPV1 channels,^54,55^ could be used to block Piezo2-mediated mechanotransduction. Alternatively, TRPV1 and TRPA1 agonists indirectly modulate Piezo2 function, as calcium influx through these TRP channels has been shown to suppress Piezo2 channel activity via intracellular signaling pathways.^51^ Among neuropeptides enriched in nociceptor-like Piezo2^+^ DPA neurons, antibodies against α-CGRP, which have shown clinical efficacy in treating headaches,^56^ may offer potential therapeutic benefits for dental pain. Other neuropeptide candidates identified in this study, including β-CGRP, substance P, galanin, and secretoneurin, may serve as novel targets for pain modulation specifically associated with mechanosensitive nociceptors.

In conclusion, our study yields significant insights into the distinct mechanosensory properties of neurons activated by Piezo2, highlighting the specialized role of mechanosensitive DPA neurons in sensory signaling. Further studies should explore how Piezo2 signaling integrates with other mechanosensory pathways to refine our understanding of its role in dental pain.

## Materials and Methods

### Animals

A total of 26 male C57BL/6 J wild-type mice (4- to 7-weeks old) (Doo Yeol Biotech, Seoul, South Korea) and 40 male and female Piezo2-ZsGreen mice (4- to 7-weeks old) were used in this study. Piezo2-ZsGreen mice were generated by crossbreeding Piezo2-EGFP-IRES-Cre mice (Cat. #027719, Jackson Laboratory, Bar Harbor, ME, USA) with Ai6 (Rosa-ZsGreen) mice (Cat. #007906, Jackson Laboratory, Bar Harbor, ME, USA), both on a C57BL/6 J background. Mice were maintained under a 12 h light/dark cycle at a controlled temperature (23 ± 2°C) and humidity (50%). All procedures were approved by the Institutional Animal Care and Use Committee (IACUC) at Seoul National University (protocol number: SNU-210125-5-6). This study conformed to the Animal Research: Reporting In Vivo Experiments (ARRIVE) guidelines for preclinical animal studies.

### Retrograde labeling

All procedures followed previously described protocols for retrograde labeling of DPA neurons.^57,58^ Briefly, the pulp of the upper molars was exposed using a low-speed dental drill under anesthesia with pentobarbital (60–70 mg/kg, intraperitoneally). Crystals of DiI (Cat. #D3911, Molecular Probes, Eugene, OR, USA) were inserted into the pulp cavities, sealed with a resin-based sealant (GC Fuji II, GC corporation, Tokyo, Japan), and maintained for two weeks before primary culture. Alternatively, for FG labeling, the dentin surface was exposed without exposing the pulp. FG crystals (Fluorochrome, Denver, CO, USA) were then applied to the dentin cavities, sealed with a resin-based sealant, and maintained for five days before primary culture.

### Primary culture of TG neurons

TG tissues were obtained from sacrificed mice and harvested in Ca^2+^/Mg^2+^-free Hanks’ Balanced Salt Solution (Cat. #LB 203-06, WELGENE, Gyeongsan, South Korea). Both TG tissues were gently dissected using micro scissors and immediately incubated in a solution containing 0.1 U /ml collagenase A (Cat. #10103578001, Roche, Risch-Rotkreuz, Switzerland) and 2.4 U/ml Dispase II (Cat. #4942078001, Risch-Rotkreuz, Switzerland) for 1 h at 37°C. TG tissues were then washed with Dulbecco′s Modified Eagle′s Medium (DMEM; Cat. #LM00105, WELGENE, Gyeongsan, South Korea) containing 10% fetal bovine serum (FBS; Cat. #16000044, Gibco, New York, NY, USA) and 1% penicillin/streptomycin (Cat. #15140122, Gibco, Waltham, MA, USA). The tissues were then mechanically dissociated using a Pasteur pipette, and the resulting cells were resuspended in Neurobasal medium (Cat. #21103049, Gibco, Waltham, MA, USA) supplemented with 1X B-27 (Cat. #12587010, Gibco, Waltham, MA, USA), 1 mM L-Glutamine (Cat. #G7513, Sigma-Aldrich, St. Louis, MO, USA), and 1% penicillin/streptomycin. The cells were cultured in a 5% CO_2_ incubator maintained at 37°C until further use in experiments.

### Immunohistochemistry

Mice were perfused with phosphate-buffered saline (PBS), followed by fixation with 4% paraformaldehyde solution (PFA; Cat. #BPP-9004, T&I, Gangwon-do, South Korea). The harvested TG tissues were transversely sectioned at a thickness of 14 µm. The TG sections were incubated in a blocking solution containing 10% normal donkey serum (NDS; Cat. #017-000-121, Jackson ImmunoResearch, West Grove, PA, USA) and 0.3% Triton X-100 (Cat. #T8787, Sigma-Aldrich, St. Louis, MO, USA) in PBS. They were then incubated overnight at 4°C with primary antibodies: mouse anti-NF200 (1:1 000 dilution, Cat. #N4142, Sigma-Aldrich, St. Louis, MO, USA) or goat anti-CGRP antibody (1:1 000 dilution, Cat. #ab36001, Abcam, Cambridge, UK) in PBS containing 1% NDS and 0.3% Triton X-100. The next day, after three washes in PBS (5 min each), sections were incubated with secondary antibodies: donkey anti-mouse Cy3 (1:200 dilution, Cat. #715-165-150, Jackson ImmunoResearch, West Grove, PA, USA) or donkey anti-goat Cy3 (1:200 dilution, Cat. #705-165-147, Jackson ImmunoResearch, West Grove, PA, USA), prepared in the same diluent as the primary antibodies. To identify cell bodies and measure their sizes, all TG sections were stained with NeuroTrace 640/660 Deep-Red Fluorescent Nissl Stain (1:100 dilution, Cat. #N21483, Molecular Probes, Eugene, OR, USA). Images were acquired using a confocal microscope (LSM700, Zeiss, Germany).

All subsequent immunohistochemical analyses and measurements of cell body area (µm²) were performed using ImageJ (NIH, Bethesda, MD, USA). Three TG sections per mouse were randomly selected for analysis. The images were converted to greyscale (8-bit), and brightness/contrast was uniformly adjusted for each antibody to ensure zero background signal. Neurons were classified as positive or negative based on whether their mean intensity value inside the cell somata exceeded a predefined threshold.

### Piezo2 knockdown and quantitative RT-PCR

Piezo2 knockdown and quantitative RT-PCR (RT-qPCR) were performed as previously described.^13^ Briefly, primary cultured mouse TG neurons were transfected with 10 nmol/L Piezo2 siRNA (Cat. #s158086, Invitrogen, Waltham, MA, USA) or 10 nmol/L scrambled siRNA (Cat. #4390843, Invitrogen, Waltham, MA, USA) 1 h after plating. Lipofectamine 2000 (Cat. #11668027, Invitrogen, Waltham, MA, USA) was used as the transfection reagent, following the manufacturer’s instructions. After 24 h of transfection, cells were used for RNA extraction to evaluate Piezo2 knockdown efficiency or for patch-clamp recordings. Total RNA was extracted using Trizol (Cat. #15596018, Invitrogen, Waltham, MA, USA), and cDNA was synthesized from total RNA using M-MLV reverse transcriptase (Cat. #28025013, Invitrogen, Waltham, MA, USA). For each qPCR reaction, 20 ng of cDNA was used, and each sample (20 μL) was run in triplicates using PowerUp SYBR Green Master Mix (Cat. #A25780, Applied Biosystems, Waltham, MA, USA) according to the manufacturer’s instructions. Primers (Cosmogenetech, Seoul, South Korea) are listed in Supplementary Table S1. qPCR was performed on a QuantStudio 3 Real-Time PCR system (Applied Biosystems, Waltham, MA, USA) under the following conditions: 50°C for 2 min, 95°C for 10 min, followed by 40 cycles of 95°C for 15 sec, 55°C for 15 sec, and 72°C for 1 min. Piezo2 expression levels were analyzed using the ΔΔCT method, with *Gapdh* as the reference gene.

### Whole-cell patch-clamp recordings

Primary cultured DPA neurons from wild-type or Piezo2-ZsGreen mice were examined under a 40X objective on a microscope (BX51, Olympus, Tokyo, Japan) equipped with infrared differential interference contrast (IR-DIC). Fluorescent signals were captured at RFP (for DiI-labeled neurons) and GFP (for ZsGreen-positive neurons) filters. A standard physiological recording solution previously described in the studies was used.^20,59^ Patch pipettes (4–6 MΩ) were pulled using a micropipette puller (Sutter P-97, Sutter Instruments, Novato, CA, USA) from borosilicate capillary glass (Cat. #BF150-86-10, Sutter Instruments, Novato, CA, USA). These pipettes were filled with an internal solution containing (in mmol/L): 123 K-gluconate, 18 KCl, 10 NaCl, 3 MgCl_2_, 0.3 GTP-Na, 2 Na_2_ATP, and 0.2 EGTA, adjust to pH 7.4 with KOH, with an osmolarity of 300 mOsm. The external solution for current-clamp experiments contained (in mmol/L): 140 NaCl, 5 KCl, 2 CaCl_2_, 1 MgCl_2_, 10 HEPES, and 10 D-glucose, adjusted to pH 7.4 with an osmolarity of 300 mOsm. For current-clamp recordings, APs were evoked by injecting 500 ms current pulses in incremental steps of 10–100 pA at 500 ms intervals.

The pipette solution for voltage-clamp for sodium current (*I*_*Na*_) contained the following (in mmol/L): 135 CsCl, 5 MgCl_2_, 5 EGTA, 2 MgATP, 10 HEPES adjust to pH 7.4 with CsOH, with 300 mOsm. The external solution contained the following (in mmol/L): 90 NaCl, 30 Choline-Cl, 20 TEA-Cl, 0.1 CaCl_2_, 5 MgCl_2_, 5 CoCl_2_, 10 HEPES, 10 D-glucose, adjust to pH 7.4 with NaOH, with 300 mOsm. Inward currents were recorded at a holding potential of -60 mV. For current-clamp experiments, voltages were recorded without injecting current during poking stimulation. In voltage-clamp experiments, *I*_Na_ was evoked by applying a test pulse to +10 mV from a holding potential of -80 mV every 15 sec. Following established protocols,^20,60^ a solution containing 1 mmol/L TTX (Tocris Bioscience, Bristol, UK) diluted in external bath solution was perfused directly onto cells using gravity-fed flow micropipettes positioned above the cell. The solution was applied for at least 1 min to ensure sufficient exposure, and *I*_Na_ was subsequently retested to confirm TTX’s ability to block sodium currents. Based on these tests, *I*_Na_ was classified as TTX-S or TTX-R. TTX-R neurons were identified when the sodium current maintained an amplitude greater than 40% in the presence of 1 mmol/L TTX. This threshold was established based on the understanding that nociceptive sensory neurons can express TTX-S sodium channels, which may result in a partial reduction in current amplitude.^24^ Patched cells were held at 0 mV, prepulsed to -100 mV for 100 ms, followed by a test pulse from -100 to 10 mV in 10 mV increments, preceded by a 0 mV step for 60 ms. Current-clamp mode for APs and voltage-clamp mode for *I*_Na_ were performed at room temperature using a MultiClamp 700B amplifier, Digidata 1550B, and Clampex 11 software (Axon Instruments, Sunnyvale, CA, USA). All data were low-pass filtered at 10 kHz and sampled at 20–100 kHz frequency. Neurons with an access resistance greater than 30 MΩ or a resting membrane potential greater than -60 mV were eliminated from further analysis. The access resistance was not compensated in current-clamp recordings, and the liquid junction potential corrections were not applied.

### Mechanical stimulation

The mechanical stimulation protocol was adapted based on the methodology of a previous study.^13^ For mechanical stimulation during whole-cell current recording, poking step indentations were applied using a fire-polished glass micropipette (tip diameter 1.5–1.7 μm). The pipette was controlled by a piezo-electric actuator (Cat. #PA50/14 SG P-155-01, piezosystemjena, Jena, Germany) and a voltage amplifier (Cat. #NV40/1 CL E, piezosystemjena, Jena, Germany). A series of mechanical steps in 1 μm increments was applied every 20 s, and each stimulus lasted for 5 ms. Mechanosensitive APs were recorded in whole-cell mode during poking stimulation.

### Single-cell reverse transcription polymerase chain reaction (scRT-PCR)

Following the completion of electrophysiological recordings, individual DPA neurons were collected into PCR tubes containing a freshly prepared collection buffer. The collection buffer comprised: 84U RNaseOUT, 2.08 mmol/L MgCl_2_, 1.67 mmol/L dNTPs, 8.33 mmol/L oligo(dT)_20_, 150 ng random hexamer, and 300 ng bovine serum albumin. RT-PCR was performed to synthesize cDNA templates. Each primer set for the target genes was designed as exon-junction primers to prevent residual genomic DNA contamination and was amplified using conventional PCR. The first-round PCR was performed in 15 μL of reaction buffer (2X Platinum Green Hot Start PCR Master Mix, Cat. #13991913, Invitrogen, Waltham, MA, USA), containing 1.8 μL of RT product and 0.2 μmol/L outer primers, followed by 20 PCR cycles according to the manufacturer’s instructions. For the second-round PCR, the reaction buffer (25 μL) contained 2 μL of the first-round PCR product, with an increase in cycle number to 35 cycles. For *Gapdh* primers, 6 μL of RT products and 0.8 μmol/L inner primers were used, following the same second-round PCR protocol. All PCR products were electrophoresed on a SafePinky-stained (Cat. # S1001-025, GenDEPOT, Baker, TX, USA) 2% agarose gel, and images were captured using a Chemi-Doc XRS+ Imaging System (BioRad, Hercules, CA, USA).

### Fluorescence in situ hybridization (FISH)

The overall procedure was carried out with minor modifications based on a previously described method.^16^ Mice were perfused with PBS, followed by fixation with 4% PFA. TG tissues harvested from the ipsilateral side of FG labeling were briefly post-fixed (less than 4 h) and subsequently transferred to a 30% sucrose solution in PBS at 4°C. The frozen TG tissues were sectioned transversely at a thickness of 14 µm. Freshly prepared sections underwent Protease IV pretreatment for 20 min at room temperature, followed by fluorescence detection using the RNAscope Multiplex Fluorescent v2 assay (Cat. #323100, Advanced Cell Diagnostics, Newark, CA, USA) with the following probes: Mm-*Scn1a* (Cat. #556181-C3), Mm-*Scn10a* (Cat. #426011-C2), Mm-*Scn11a* (Cat. #403531-C2, Mm-*Trpv1* (Cat. #313331-C3), Mm-*Calca* (Cat. # 578771-C3), Mm-*Calcb* (Cat. #425511-C3), Mm-*Tac1* (Cat. #410351-C3), Mm-*Gal* (Cat. #400961-C3). All z-stack images were acquired using a confocal microscope controlled by ZEN software (LSM900, Zeiss, Germany). FG signals were captured in the DAPI range, while probe signals were detected using the Cy3 filter cube (for C2) or the Cy5.5 filter cube (for C3).

Confocal images were analyzed to measure cell body area (μm^2^) of FG-labeled DPA neurons and the average intensity of fluorescent puncta within neurons using Image J. Three to four TG sections per mouse were randomly selected, and the images were converted to greyscale (8-bit) with equal brightness/contrast adjustments applied for each probe to ensure a zero-background signal. DPA neurons were considered positive if they contained ten or more puncta of the amplified probe signal or if the average intensity exceeded 10% of the maximum value.

### Differential expression (DE) analysis

We conducted secondary analyses on the dataset originally sourced from a previous study.^16^ The DPA neuron transcriptome read counts were log-normalized and center-scaled for reanalysis in this study. For DE analysis, read counts were normalized for library size using the DESeq2 package in R. Volcano plots showed enriched DE genes in each group with fold change (log_2_ scale) > 1.0 and an adjusted *P*-values < 0.05.

### Correlation analysis

Pearson correlation coefficients (R) and *P*-values were calculated for all FG-labeled DPA neurons acquired from FISH images (Fig. 5b, c, f) and visualized using the ‘ggpubr’ package in RStudio (version 4.2.3).

### Data analysis and statistics

Bar graphs are presented as mean ± SEM. The differences between two groups were compared using a two-tailed Student’s *t*-test, Welch’s *t*-test, or Mann-Whitney *U*-test, conducted with Prism software (version 8.00 or 10.00; GraphPad, Boston, MA, USA). Comparisons among three groups were analyzed using a one-way analysis of variance (ANOVA) followed by Bonferroni’s or Dunn’s multiple comparisons test, or Kruskal-Wallis test. Differences were considered statistically significant at *P*-values < 0.05.

## Supplementary information

Supplementary figures and tables

## Data Availability

All data generated or analyzed are presented in this article and its supplementary information files.
