# Supplementary material for: Functional and distinct roles of Piezo2-mediated mechanotransduction in dental primary afferent neurons
Source: Int J Oral Sci. 2025 Jun 3;17:45. doi: 10.1038/s41368-025-00374-8 (PMC12134352; doi:10.1038/s41368-025-00374-8)
Supplement: Supplementary file 1 — Supplementary figures and tables [file 41368_2025_374_MOESM1_ESM.pdf]

Supplementary Information

Functional and distinct roles of Piezo2-mediated mechanotransduction in dental primary afferent neurons

Pa Reum Lee[1,2], Kihwan Lee[3], Ji Min Park[1], Shinae Kim[1], and Seog Bae Oh[1,3,4]

[1] Department of Neurobiology and Physiology, School of Dentistry and Dental Research Institute, Seoul National University, Seoul 03080, Republic of Korea

[2] Brain Science Institute, Korea Institute of Science and Technology, Seoul 02792, Republic of Korea

[3] Tooth-Periodontium Complex Medical Research Center, Seoul National University, Seoul 03080, Republic of Korea

[4] ADA Forsyth Institute, Cambridge, MA 02142, USA

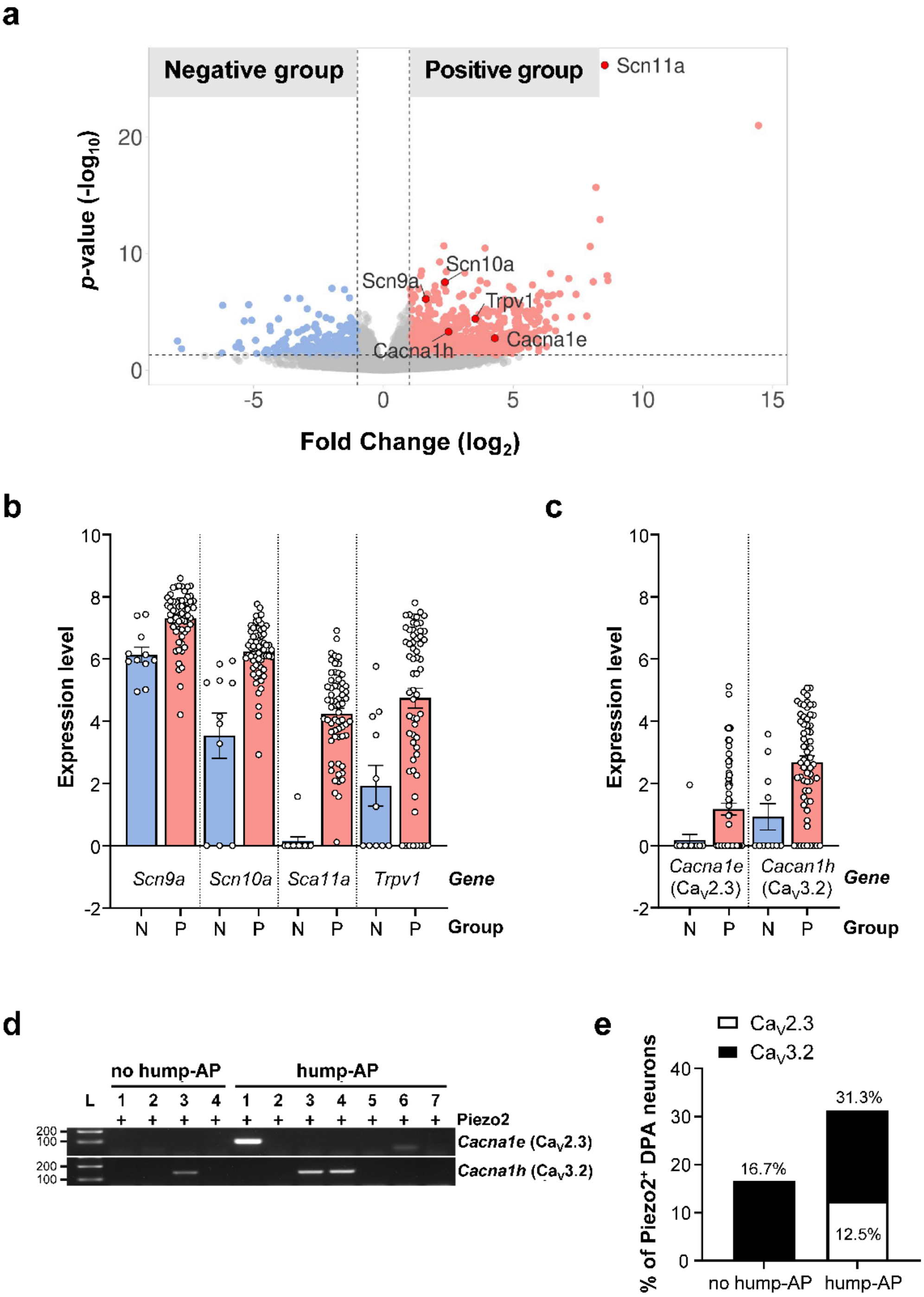

Figure S1. Expression of voltage-gated calcium channel genes upregulated in the positive group of Piezo2-ZsGreen$^+$ DPA neurons.

a Volcano plot showing DE genes with a $\log_2$ fold change > 1.0 and an adjusted *p*-value < 0.05 (dotted lines), enriched in the negative group (left side) or the positive group (right side) within

Piezo2+ DPA population. Genes of interest are labeled in the plot. **b** Log-normalized expression levels of *Scn9a*, *Scn10a*, *Scn11a*, and *Trpv1*. **c** Expression levels of voltage-gated calcium channel genes, *Cacna1e* ($Ca_V2.3$) and *Cacna1h* ($Ca_V3.2$). **d** Representative gel images of scRT-PCR products from no hump-AP and hump-AP neurons. Predicted sizes for selected genes: *Cacna1e* ($Ca_V2.3$): 108 bp; *Cacna1h* ($Ca_V3.2$): 145 bp. The same cDNA as Fig. 4a was used. **e** Summary of scRT-PCR results for $Ca_V2.3$ and $Ca_V3.2$ in no hump-AP ($Ca_V2.3$: 0%, n = 0/6 neurons; $Ca_V3.2$: 16.7%, n = 1/6 neurons) and hump-AP neurons ($Ca_V2.3$: 12.5%, n = 2/16 neurons; Cav3.2: 31.3%, n = 5/16 neurons). Data represent results from n = 8 mice.

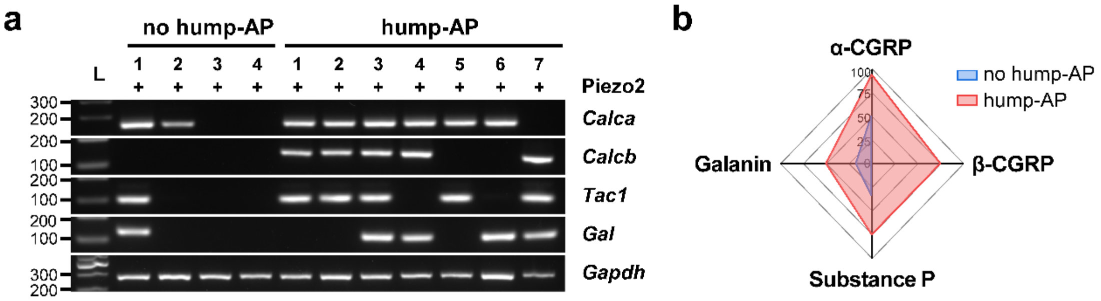

Figure S2. Neuropeptide expression in no hump-AP and hump-AP Piezo2-ZsGreen⁺ DPA neurons.

a Representative gel images showing scRT-PCR products from no hump-AP or hump-AP Piezo2-ZsGreen+ DPA neurons. Predicted sizes for selected markers: *Calca*: 156 bp, *Calcb*: 153 bp, *Tac1*: 100 bp, *Gal*: 127 bp, and *Gapdh*: 282 bp. Gene names are indicated by their *gene symbols*. The same cDNA used in Fig. 4a was used for this experiment. **b** Radar plots summarizing the proportion of each neuropeptide gene expressed in no hump-AP and hump-AP neurons, respectively.

Table S1. List of primer pairs used.

Target gene	scRT-PCR		GenBank No.
	Outer primers	Inner primers	
Gapdh<br>(282 bp)		(F) CCAGAACATCATCCCTGCAT<br>(R) GCATCGAAGGTGGAAGAGTG	NM_001289726.2
Trpv1<br>(293,115 bp)	(F) CATCTTCACCACGGCTGCTTA<br>(R) AGAACACCATGGAAGCCACAT	(F) GCGACCATCCCTCAAGAGTT<br>(R) ATACTCCTTGCGATGGCTGA	NM_001001445.2
Scn1a<br>(357,123 bp)	(F) CACAAGTGGCATAGGGACGG<br>(R) CCTCTTCCTTCTTCCACGCTG	(F) GGAACAGCCTGTCATTGAACC<br>(R) CACCACTGTTTCCCTCTTCCT	NM_001313997.1<br>NM_018733.2
Scn8a<br>(222,97 bp)	(F) GCCATGTGCCTTATCGTGTT<br>(R) CTTGAAGTGTGCCTGCATGA	(F) CAACCTGGTGGTGCTGAACT<br>(R) GTTGTTCATTTCCCCGTCGT	NM_001077499.2<br>NM_011323.3
Scn9a<br>(313,144 bp)	(F) GAATTCACCTTCCTCCGTGAC<br>(R) CATTCTGCTCAAGGTCCTTCC	(F) ACTGGCTGGACTTTGTTGTC<br>(R) CCCCCACGATGGTTTTTAGTC	NM_001290674.1<br>NM_001290675.1
Scn10a<br>(311,190 bp)	(F) GCCACTTCTTCTGGGTTAACG<br>(R) GTACTTCTTCTGCTCCTCTGTCAT	(F) CGTTGCTATGGGCTACCTCG<br>(R) CCCGACAAAGAGATTCAGCG	NM_001205321.1<br>NM_009134.3
Scn11a<br>(349,209 bp)	(F) ACCACGGGGTTGAAATGGAA<br>(R) TCGAATATCAGTGCTCCGCT	(F) CCATACAGAGTGCTCGAAAGAAG<br>(R) AAGATTCCACCACAGGACCC	NM_011887.3
Tyrosine hydroxylase<br>(317,152 bp)	(F) ATACAAGCAGGGTGAGCCAAT<br>(R) AGGCATGACGGATGTACTGTG	(F) GTACTGTGGCTACCGAGAGGA<br>(R) TGCACTGAAACACACGGAAGG	NM_009377.2
Calca<br>(242,156 bp)	(F) GCCTTTGAGGTCAATCTTGG<br>(R) CCTTCACCACACCTCCTGAT	(F) CACTCTCAGTGAAGAAGAAGTTCG<br>(R) GTCACACAGGTGGCAGTGTT	NM_001033954.3<br>NM_001289444.1
Calcb<br>(313,153 bp)	(F) CTCTCAGCAGCATATGGGTC<br>(R) ACATCGGTGGGCACAAAGTTG	(F) CTTTGGAGAGCAGCCTAGAC<br>(R) TCTTCTGAGCAGTGACACGG	NM_054084.2<br>NM_001429730.1
Tac1<br>(218,100 bp)	(F) CATGAAAATCCTCGTGGCCG<br>(R) CATCCCGCTTGCCCATTAATC	(F) CAGAGGAAATCGATGCCAACG<br>(R) AGAAGATGCTCAAAGGGCTCC	NM_009311.3<br>NM_001311060.2
Gal<br>(300,127 bp)	(F) GAGGCAGCGTTATCCTGCTA<br>(R) GGCCTTTAAGGTGCAAGAAACT	(F) AGAGAGGTTGGACCCTGAACA<br>(R) CTTTCCTCCACCTCCAGTTGTAA	NM_010253.4<br>NM_001329667.1
Cacna1e<br>(327,108 bp)	(F) TTTGTACAACCCCATACCAGTCC<br>(R) CAGCCATTACGGAGGTATGAAC	(F) GATGACAAGACCCCAATGTCTC<br>(R) GAAGATGAACCCTAGAGCCACA	NM_009782.3
Cacna1h<br>(302,145 bp)	(F) GGTTTGTAACCCGTGCTTCGAG<br>(R) CAGAGAGGCTCACGTTGTGTC	(F) CATCTTGGAGGCCTTTGATGACTT<br>(R) GCCAGCCATGACAATGAAGAAGT	NM_021415.4<br>NM_001163691.1
	**Quantitative RT-PCR**		
Gapdh<br>(282 bp)		(F) CCAGAACATCATCCCTGCAT<br>(R) GCATCGAAGGTGGAAGAGTG	NM_001289726.2
Piezo2<br>(160 bp)		(F) ATTGCTGGCAATGACACAGA<br>(R) TGTCTCTGAACAAAATGATGGTG	NM_001039485.5

Table S2. DE genes upregulated in the negative group, related to Fig. 7a–b (sorted by descending $\log_2$ Fold Change).

Gene Symbol	Base Mean	$\log_2$ Fold Change	$-\log_{10}$ Adjusted *p*-value
Pcp4	645.3389506	6.192442153	3.271834655
Me3	11.35627742	5.358404386	2.284228546
Fhod3	31.72263304	5.186499735	3.311180488
Baiap2l1	15.66735219	5.074356943	2.331805038
Sparcl1	76.40796408	4.373348252	1.986508277
Ano4	31.87941822	4.349081031	1.72438378
Tesc	29.81843085	4.03751937	2.426811496
Osbp2	17.87613713	3.691747807	3.73021234
Ripply2	2.877659595	3.65783726	1.627784511
Rassf8	7.380365035	3.30647553	1.627845306
Nms	23.68245342	3.273085333	1.645445458
Car2	487.4441123	3.215894117	3.714340101
Mapk4	18.69229921	3.106330672	1.771840817
Spink2	11.52028576	2.949679808	1.906340167
Sncb	10.67126103	2.930197279	3.024210249
Arap2	69.02636357	2.777682477	1.330615079
Vsnl1	19.84094744	2.655007408	1.952590062
Star	16.56150933	2.62133386	1.353739897
Mest	53.32499922	2.618184291	2.084376188
Crip1	7.333347871	2.551449556	1.788520168
Rgcc	18.58663744	2.495172064	1.603533534
Elmo1	67.81804565	2.48412584	2.687590592
Trib1	17.63776571	2.24419952	1.386720235
Arid5b	56.77494841	2.205954304	2.491294781
Mtfp1	19.0816535	2.088277187	1.512676132
Osbpl1a	77.89087015	2.01330781	2.393058276
Adarb1	61.4075124	1.977169067	4.256314584
Kcnc3	5.265352111	1.864099834	1.393495041
Nsg1	138.3659706	1.733062625	1.425707023
Zfp385b	108.3379358	1.676174445	1.986508277
Rcan2	707.9118958	1.645476849	1.580655977
Nfkb1	30.5788613	1.624098464	1.330615079
Cdr1	1270.703735	1.622555313	1.965069745
Ints9	123.2613539	1.578560861	1.613685304
Ldhb	928.1188187	1.495215125	4.202092914
Ckmt1	533.5164523	1.39659216	1.735635298
Vamp1	572.5272972	1.391730958	2.051108863
Cplx1	1995.029353	1.302821786	3.753308255

Podxl2	63.94749338	1.283704858	1.678946218
Ell2	185.8942743	1.264168489	2.48453188
Onecut2	359.1734156	1.24600142	1.974284207
Cds1	115.7122298	1.242123242	2.447426937
Snhg8	19.43006353	1.217316649	1.974284207
Fabp3	178.1628232	1.209438598	1.778968022
Gadd45a	544.271345	1.200841318	1.302170999
Haghl	174.9043048	1.198169297	2.297216863
Mark1	160.6177556	1.196987075	1.699995622
Szrd1	18.94545189	1.140854084	1.655399669
Nefm	2215.992635	1.121592763	1.678946218
A830018L16Rik	334.6424811	1.106014677	1.94728042
Nefl	6160.129879	1.097776104	2.087799667
Nat8l	172.4706393	1.062006225	2.110163103
Aebp2	90.04479507	1.011976806	1.391332729
Stx5a	221.9568897	1.008363819	1.723340309

Table S3. DE genes upregulated in the positive group, related to Fig. 7a–b (sorted by descending $\log_2$ Fold Change).

Gene Symbol	Base Mean	$\log_2$ Fold Change	$-\log_{10}$ Adjusted *p*-value
Trpa1	1225.096742	14.45639808	17.11791167
Cfh	53.07490271	8.652864208	4.733793152
Gm7271	52.21451262	8.629131869	5.093831759
Scn11a	125.0902171	8.532356635	21.99444503
Kcnt2	43.15525316	8.354058077	9.34538774
Calcb	460.0520791	8.193791914	11.981414
Trim30d	35.9017317	8.088618358	4.675970907
Casp1	147.9131038	7.971306005	7.214797582
Inhbb	15.98440147	7.840610995	2.587669317
Sp100	22.66894089	7.425457069	3.931921052
Ndnf	19.92271044	7.239648952	2.519167318
Rgs14	18.69062844	7.147859729	4.866586876
Zfp300	25.37610876	6.799145237	3.940752472
Clca5	13.11331648	6.636876249	1.719806333
Rgs8	27.79342385	6.626825766	2.50029867
Ncapg	12.22354831	6.535224393	3.920048772
Vdr	11.57410024	6.456312268	3.870109521
Aldh1a3	160.4150165	6.439801833	5.205488129
Cd28	10.93150335	6.372485233	1.879125836
Zkscan4	10.74591001	6.348330128	3.391582868
Serpina3c	10.49556276	6.315504608	3.870109521
Psg16	10.21429707	6.276430096	2.235169373
Fam84b	9.971440592	6.24026006	1.855018362
2610018G03Rik	9.724458452	6.203963908	1.778628203
Galr1	9.697127776	6.199854846	1.725628007
Kcnn2	22.42961716	6.089038165	3.986660535
Fam211a	8.928497864	6.082275352	4.002403663
Samsn1	12.46790412	6.032397548	3.191785684
Pcdhb18	8.522001227	6.014887214	1.738931905
Car12	16.98708583	6.008946921	2.474001039
Casr	8.453118301	6.002943925	2.931393751
6030408B16Rik	80.42538667	5.983086557	3.889632786
Rhpn2	8.211441029	5.958139682	2.242474218
Kcng2	9.567091189	5.915431195	3.58633877
Il33	39.24302249	5.909908736	2.621438561
Rassf7	7.510121306	5.833506478	2.077575411
Topaz1	9.007776678	5.831511753	1.610916212
Ccl7	7.360393905	5.803196163	1.404782946
Megf6	7.163063049	5.765092287	2.943112317

Fmo5	15.0353403	5.749064569	4.463974657
Chia	6.97939492	5.728223313	2.834430433
Col25a1	6.96052114	5.720826983	1.391338839
Kcng1	9.892230975	5.702057152	2.235169373
Ifi47	15.63075448	5.65070732	2.498152714
Gpr119	6.585178143	5.643633507	1.519437393
3425401B19Rik	6.602396095	5.643631211	2.424689666
9330179D12Rik	6.494722828	5.621986946	1.398317744
Bmpr1b	11.10929168	5.608463429	2.669209421
Dpp4	6.290912056	5.573903036	2.722308973
Apol9b	6.278982949	5.573742812	2.491791182
Flrt2	6.210962322	5.558624249	1.422944422
Aqp1	60.69033808	5.533950709	2.893104118
Krt79	6.004242498	5.508576934	1.922872944
Ndc80	5.975247341	5.503723048	1.33438575
P2ry14	16.12194089	5.499415642	2.687587392
9830147E19Rik	12.36059118	5.468022244	1.9063451
Gm6416	10.03413441	5.459312958	1.740267277
Stil	5.688034539	5.432321602	1.646046405
Mcmdc2	5.652504745	5.419858893	1.362857926
C1qtnf3	5.386078424	5.351067253	1.9063451
Kcnc4	11.41388659	5.341125904	1.759134465
Efcab11	5.173438057	5.296095603	2.931718812
Frrs1	8.863811536	5.282451177	3.310588961
Insrr	7.41110129	5.275430588	2.017156688
Ly75	5.074548987	5.259176794	3.124368187
Bdkrb2	5.027915077	5.256237628	1.761667701
Grhl2	5.027192522	5.254055852	1.397918739
Mki67	5.015337328	5.249816567	1.743859076
Pdgfra	5.981679131	5.237884361	1.690903471
Ak4	4.922622394	5.224458061	1.464282326
1600029O15Rik	7.041494817	5.209888471	2.249672677
Catsperd	4.831843567	5.192621079	2.520881234
Serpinb5	20.3676508	5.173752162	2.633261308
Gsg1l	8.155108637	5.159154939	1.472539503
Snora28	4.676532634	5.146248294	1.98724863
Thbd	54.43705397	5.133707831	3.455791205
Dnm3os	4.625302349	5.131547834	1.305799919
Trpc7	4.590331927	5.125938223	1.833342193
Tmem232	4.597603724	5.122691879	1.353735394
Slfn8	12.32272599	5.103473222	2.010798582
Aim1	7.815878083	5.096143767	2.331799899
Comp	5.408901003	5.091058037	2.192537515
C530044C16Rik	7.694904868	5.072216303	1.384082788
Hmgn5	7.60297818	5.054708153	2.798675306
Cxcl1	6.248164391	5.035832871	1.678944159

Mnda	11.51815484	5.029280589	3.017420751
Pdgfb	4.295096072	5.028477064	1.568308801
Zfp442	27.13492526	4.988933985	4.233287821
Hoxd1	36.03032203	4.984706588	3.603796544
Ssc5d	4.145267797	4.978075741	1.425710881
Bmper	29.02102822	4.962752868	3.259036775
Celf6	41.887742	4.90755441	3.375261624
Morc1	3.864077513	4.871772157	1.610916212
Trps1	19.29388398	4.869857462	3.391582868
Trim12c	32.5739086	4.862711586	4.391183398
Slc35g1	8.445419786	4.827618267	1.587436988
Smpd5	6.212693806	4.763201585	1.548127723
Gnat1	3.585902076	4.761790815	1.390317317
Rad9b	10.26528083	4.678954885	3.66717139
Ptprt	7.190791925	4.673081984	1.459911141
Nat2	19.69644828	4.654953731	2.461501966
9130017N09Rik	8.696979574	4.653187696	3.651384082
Npy1r	189.2674316	4.649864133	2.217753859
Gm16702	5.632010615	4.634161775	3.117277049
Gria1	16.2864865	4.590612749	3.391582868
Gramd2	3.79592387	4.584074242	1.321107351
Prickle1	8.93649519	4.581413838	2.21195971
Prr16	3.108820978	4.557283925	1.593350635
2010002M12Rik	26.58414057	4.512792391	2.970522114
Disc1	2.951949576	4.491697699	1.965072401
4833428L15Rik	2.952194273	4.48473914	1.862321881
Ccl2	182.6610583	4.478921365	2.225309521
2310030G06Rik	6.837279394	4.44860315	1.390425787
Abhd15	4.158631693	4.448415896	1.357510015
Gal	182.4176558	4.434847169	1.976498096
Ly6e	79.52785325	4.43030765	2.654099038
Apln	5.843282008	4.40470022	2.037305969
1810024B03Rik	5.787037846	4.378208432	1.94012867
Dlx3	4.521753467	4.302095402	1.811647192
Cacna1e	10.27198864	4.298954027	1.304529194
Tmtc2	16.59231183	4.250860121	2.876466065
4930429F24Rik	12.85062762	4.19434187	1.421720796
Igj	4.134318229	4.171366586	1.965072401
AW146154	25.33874034	4.166828642	2.873509631
Hrh1	15.62021464	4.147048986	1.992318121
Gm2518	2.27091622	4.115983567	1.512677932
Apobec1	9.133795684	4.115344305	1.450507348
Avpr1a	51.59290699	4.056922047	1.971870651
A2m	16.81386445	4.037975437	2.580175748
Rnf182	10.25898196	4.007615193	2.931718812
Rgs4	2003.693676	3.994659107	4.573138982

Fam3b	4.455400116	3.97065066	1.533948273
L3mbtl1	7.249456136	3.964729261	3.009643592
Hapln3	7.640923542	3.957294673	1.993185751
Sdk1	2.437269823	3.949297528	1.655396258
Fam89a	180.6573697	3.919480794	7.14180931
Mal2	374.9663577	3.882750785	2.873509631
Lhfpl1	130.3253704	3.85356257	2.808792059
Sp110	12.81819208	3.836783877	2.893104118
Trhde	45.66956156	3.809546591	2.011056039
Zfp947	40.61701026	3.794205532	2.154502283
Rex2	2.205009418	3.781614	1.811004134
2310042D19Rik	2.653914008	3.777730323	1.645448725
Rnf165	2.154270592	3.765693388	1.355870873
C1qtnf7	165.4228927	3.734107449	4.733793152
Scube1	6.866941619	3.727574413	1.391338839
1700001O22Rik	1.729393444	3.712862425	1.305764491
Flt3l	6.565323098	3.684427909	2.322323423
Tac1	4696.422754	3.678154638	2.687587392
Bcl2l11	7.40145142	3.669295416	1.464282326
Prima1	4.789748923	3.652443201	1.807209666
Atp2b4	126.1904642	3.645018447	4.1635703
Pbld1	1.717800097	3.640503328	1.832146252
E430018J23Rik	10.08522911	3.554620042	2.420698871
Trpv1	463.4516983	3.541926536	2.428976773
Prdm8	11.50033351	3.451953304	3.872408274
Ddx60	88.89496116	3.440167254	1.760376667
Matk	2.870689735	3.414902881	1.384082788
Igfbpl1	11.09008844	3.38447205	1.397908459
BC028528	32.5489213	3.247199686	2.08110979
Sulf1	51.77618501	3.224757703	2.428475813
Thsd1	4.584737957	3.218202208	1.302173064
Trabd2b	29.4483851	3.210645221	2.432236726
Col24a1	120.7119223	3.206152484	2.63933443
Kit	30.50238734	3.201901253	1.304521213
Ifit1	793.0701285	3.187543679	2.582170583
Kcnd3	76.25240458	3.183893473	2.879955481
Tifab	7.757871889	3.183712628	1.703367991
Baalc	15.71875304	3.137937183	5.222273289
Trim68	26.42545724	3.10342667	3.002903361
Lime1	18.21545647	3.074884986	3.931921052
BC030867	50.29305115	3.057550514	3.106288288
Adcy7	107.6072007	3.011178162	2.08110979
2810454H06Rik	5.057785405	3.009832944	2.21195971
Bai3	17.58164447	3.006948522	1.347956589
A130049A11Rik	6.255062441	2.976057579	1.413084496
Slc45a3	40.72387378	2.975161936	2.621438561

Ntsr2	19.5379774	2.961152524	2.133933464
Chp2	185.1099644	2.937845163	3.849471892
Tenm1	36.40571691	2.931244636	1.986516032
Ttc38	7.778129002	2.928214	1.966395247
Ptprk	17.16112832	2.920976746	1.9063451
Gm4013	2.084284212	2.880875072	1.305799919
Cldn9	28.26180441	2.861770954	1.392010187
Kcnmb1	209.8630752	2.861485878	2.165778911
Galnt7	14.05585251	2.849389756	1.926198754
Unc13b	20.66838004	2.824317409	2.345358255
Oprm1	13.16322642	2.808017191	2.474001039
Sp6	32.27458535	2.783547155	1.952585287
Incenp	9.175885611	2.767671784	2.31540836
Adamtsl3	7.945300806	2.753062583	1.685156423
Stom	16.8547921	2.706643978	1.527062369
Nmb	744.8875244	2.689668669	2.931718812
Gm9776	14.93939199	2.679477642	1.699481923
Zdhhc12	36.85640578	2.639804169	1.944917352
Gm12942	22.31746782	2.604831741	2.707811409
Trim34a	134.0330409	2.583250226	1.767721138
Cpne4	44.33551171	2.562691622	2.474001039
Pdia5	27.0530607	2.514095218	1.568308801
Cacna1h	32.02230582	2.512886354	1.661499218
Plcg1	11.13030136	2.498718356	1.675391721
Zfp354c	38.46085897	2.497834738	1.992318121
Radil	17.38754727	2.465854142	1.788522404
Ttc12	19.1784153	2.456216831	1.304521213
Kif26b	12.04859121	2.438930021	1.492612832
Znf512b	13.4313123	2.431176421	2.331799899
Epha5	167.0157638	2.427442293	5.281623801
Ldlr	105.1443421	2.409439734	4.118636562
Fam188b	23.39986015	2.403900978	2.558112081
Zfp282	10.74281293	2.3857135	1.570817032
Ccdc60	4.61171886	2.382430448	1.303590433
Scn10a	601.5436753	2.37440557	4.664836266
Tmem176a	1710.737308	2.338186595	4.391183398
Nxn	28.89353408	2.335140684	2.431560784
Grn	159.4197774	2.33405173	7.214797582
Sirpa	50.91210198	2.318286067	1.901583211
Calca	62255.44557	2.300565112	2.165778911
Nckap5l	9.696300557	2.300013462	1.645448725
Far2	22.66348887	2.281990467	1.986516032
Irf7	77.50612263	2.275936144	1.929638789
F2r	808.6051227	2.274107922	1.393505202
Aars2	31.02011596	2.245280614	1.743859076
Tmem173	210.7367933	2.230179334	1.986516032

Ankrd6	117.4114515	2.21528319	1.952677896
Nptxr	6.684185097	2.213351066	1.711818244
Pear1	56.0468709	2.208588094	1.961108926
Pmp22	2727.171652	2.173011607	6.008103974
Timeless	183.6696585	2.157724042	3.399002044
Fam175a	43.0203871	2.139792654	1.357510015
Exoc8	59.38441885	2.131060166	3.689777644
Dyrk3	32.9937258	2.109197701	1.634698334
Nrp1	299.3877735	2.092480085	3.967760403
Tmem158	111.8943516	2.091548711	1.335843488
Pced1b	4.869990613	2.08162619	1.75630435
Slc16a2	49.26619227	2.064558153	1.376566858
Klhl32	25.98355408	2.054419847	1.471373473
Tmem176b	3271.661111	2.03108412	3.931921052
Klrg2	25.56169258	2.003892763	2.010261692
Arxes1	514.0513156	1.998823276	2.722308973
Disp2	256.7672097	1.996577617	4.413472291
H2-M3	37.96965526	1.97621122	1.749572696
Kcnb1	90.62563691	1.961979772	4.120478539
Syt7	24.68781093	1.961799464	1.745471345
Ecm1	114.3332614	1.960918108	1.486941496
Nptx2	290.5145312	1.949852658	3.633837603
Snca	317.0890827	1.918094625	1.690903471
Phf11b	47.93996399	1.916631676	1.389159966
Dsel	168.7950783	1.901791086	2.040338424
Mfsd10	67.1188286	1.893159993	3.66717139
Scg2	3902.631556	1.888988904	2.687587392
Cacna1c	14.47640114	1.862098474	1.765460758
Lgals9	911.8661116	1.857636976	3.683256319
Sema6d	90.6702657	1.849498803	2.808792059
Dip2a	12.11020567	1.844268825	1.409423462
S100a4	43.77325953	1.82260444	1.591658773
Pofut1	19.1602919	1.822536151	1.612943729
Rhbdd1	60.99066679	1.81240735	1.527323055
Tacc3	19.26026594	1.808776707	1.62909482
Plekho2	68.58525552	1.797297983	2.97397495
Vmac	29.75217035	1.791205697	1.819906974
Lst1	6.974004527	1.785750842	1.749213117
Myo1b	111.9983424	1.782818258	2.24030044
Klhl18	11.415292	1.777485395	1.874745009
Dgcr8	24.30441398	1.767648905	1.519437393
Slc7a4	69.22272131	1.759693916	1.813465673
Fam210b	158.6099747	1.747994908	1.467206861
Ncor2	35.43611513	1.742031831	2.931718812
Endov	21.04978509	1.730212362	2.331799899
Ptgir	505.2498064	1.728800433	2.04718664

Stat2	89.24488818	1.724943737	3.311185408
Cnih2	90.60157132	1.722167112	1.357477556
Dagla	31.55097755	1.718284032	2.519002939
Slc22a15	39.59686082	1.712238406	1.690903471
Kdelc2	31.87912496	1.703911441	1.330611385
Hist1h2ac	80.38538552	1.702001397	1.355870873
Arxes2	2902.381327	1.700566398	2.195703245
Ngfr	580.0595062	1.698057008	3.931921052
Stx1a	158.5338463	1.68416177	3.931921052
Sdc3	70.94729291	1.668209963	1.398317744
3110047P20Rik	129.347392	1.659538703	1.536455708
F2rl2	668.3404274	1.655357867	2.145032935
Mrgpre	68.66127632	1.638535043	1.879125836
Scn9a	1762.88163	1.636225446	3.689777644
Kcnd1	106.5234626	1.613644425	2.312806896
Abcc4	81.31475192	1.609261128	2.012704123
Crlf2	14.05182523	1.592375753	2.621438561
9530059O14Rik	47.78678353	1.588599115	2.165778911
Zfp2	136.4328818	1.586565672	1.519250712
Psme1	88.86487499	1.558229896	1.3942709
Rgs3	54.46655689	1.547086233	1.940280895
Cmpk2	59.7491732	1.539102721	1.309542961
Pstpip1	121.9377964	1.522757702	1.499143657
Cyp2j6	193.0434828	1.522405297	1.398317744
Gm4944	280.4290085	1.502968113	1.392010187
Tspan6	204.853864	1.489051065	1.838607086
Plcb3	140.3105219	1.475687653	1.561842759
Cers4	395.9583944	1.474279189	5.299956338
Sumf2	23.70576351	1.451487239	1.384082788
Paqr7	38.5849614	1.451095491	1.390317317
Lcorl	151.3963323	1.446666915	1.307974651
Sorbs1	270.0205115	1.441442817	5.093831759
Gpd2	76.57849389	1.434102961	1.596833404
Myadm	379.6300796	1.430488328	2.165778911
Bcl6	36.27334797	1.406424008	1.762460975
Prkcd	533.9842445	1.406011529	1.711899585
Rgs11	61.29870326	1.404949164	1.982188375
Man1c1	76.02023825	1.385535819	1.587436988
Map9	171.2637566	1.383287234	3.052323604
Itga3	67.06230562	1.381088115	1.561842759
Serpina3n	1293.529924	1.379978382	2.759877082
Fasn	229.4746097	1.377454222	1.92902539
Lpcat3	106.7326991	1.36954285	2.073301239
Atp1b3	2014.009874	1.363973826	4.238667818
Ptprg	187.0652981	1.360919339	1.855018362
Sprn	254.1778165	1.360843806	2.956005173

Marveld1	92.84991684	1.351107041	1.305764491
Kcnb2	74.18955328	1.339872548	1.855018362
Slc39a11	54.56029369	1.335197263	1.357510015
L1cam	258.7030785	1.31514951	2.331799899
Pirt	2014.768491	1.312221243	1.9063451
Plxna4	254.2566843	1.29903427	1.591658773
Rrbp1	47.48113609	1.295083208	1.986516032
Grm7	116.099773	1.295008401	1.397918739
Magt1	156.5675505	1.285690735	1.992318121
Bdnf	992.9559866	1.280840219	1.9063451
Cdkn1b	22.02957686	1.273960852	1.321107351
Tspan2	400.134036	1.273482664	1.62909482
Mitd1	138.3360041	1.245271017	1.649304338
Camk2d	1465.656487	1.244790581	1.568308801
1700019D03Rik	75.06316356	1.239359434	1.527062369
Hif1a	503.9029425	1.23800839	1.808575819
Pls3	1447.3063	1.22896993	3.792225821
H2-D1	811.8831307	1.225788667	1.91989889
Atp8a1	457.7227888	1.22415077	1.540767296
Ddx58	147.0175195	1.213005104	1.397908459
C1galt1c1	391.449227	1.206598164	2.225882807
Celf4	763.990917	1.19209722	2.21195971
Pigz	191.5142276	1.190105273	2.165778911
Sigmar1	638.9100787	1.178838264	3.931529754
Zdhhc20	197.2569894	1.176019407	1.655396258
Slc44a2	154.1647242	1.160769332	1.390317317
Tmem120b	89.99903397	1.153328753	2.064389184
Pvrl3	680.5315394	1.138472944	1.958782663
Sf3a1	82.89773904	1.125902577	1.684703115
Tmem237	85.72043548	1.119651189	2.955386012
Ctsl	4434.795507	1.115538912	3.476470027
Plod3	109.3432981	1.111349705	1.535279541
Slc7a7	385.2551414	1.105558741	1.376180483
Kdsr	158.9285788	1.104293316	3.741356636
Magee1	523.5406591	1.100454353	2.08110979
Gpd1l	105.6496749	1.091109852	1.858529079
Slc2a3	324.042242	1.088568918	1.502310839
Slc2a6	255.3336601	1.074257715	1.618585352
Lgmn	1520.043472	1.071381855	3.430078636
Wbscr17	49.06729996	1.071195965	1.400288076
H2-DMa	136.2225907	1.069912925	2.284970367
Idh1	1070.639355	1.069170684	2.468307973
Gpr149	274.1791011	1.065768938	1.626731314
Amigo1	271.658884	1.06286063	1.71132174
Syngr3	1478.099572	1.061383888	3.874949349
Dhcr24	212.3082523	1.052882038	1.37984564

Cyb561	432.1006946	1.042529135	1.384828178
Cyb5r1	1444.362511	1.035917908	4.239599135
Lrrc8d	233.8995492	1.028789567	1.940280895
Oasl2	875.0966418	1.019700052	2.054533589
Tmem150c	58.76163679	1.017635682	1.322383777
1700011J10Rik	99.0230304	1.012945845	1.871472617
Cyp51	415.4615454	1.011362963	1.587436988
